# Melanophilin-induced primary cilia promote pancreatic cancer metastasis

**DOI:** 10.1038/s41419-025-07344-2

**Published:** 2025-01-16

**Authors:** Yu-Ying Chao, Ruei-Ci Lin, Ping-Jui Su, Chu-An Wang, Ting-Yuan Tu, Ya-Chin Hou, Yi-Tzui Tsai, I-Chen Peng, Shaw-Jenq Tsai, Yan-Shen Shan, Chia-Yih Wang

**Affiliations:** 1 Institute of Basic Medical Sciences, College of Medicine, National Cheng Kung University, Tainan, Taiwan; 2 Department of Cell Biology and Anatomy, College of Medicine, National Cheng Kung University, Tainan, Taiwan; 3 Division of General Surgery, Department of Surgery, National Cheng Kung University Hospital, Tainan, Taiwan; 4 Department of Biomedical Engineering, National Cheng Kung University, Tainan, Taiwan; 5 Institute of Clinical Medicine, College of Medicine, National Cheng Kung University, Tainan, Taiwan; 6 Department of Physiology, College of Medicine, National Cheng Kung University, Tainan, Taiwan; 7 Department of Life Sciences, National Cheng Kung University, Tainan, Taiwan; 8 Department of Surgery, College of Medicine, National Cheng Kung University, Tainan, Taiwan

**Keywords:** Pancreatic cancer, Organelles, Cilia, Metastasis

## Abstract

Pancreatic ductal adenocarcinoma (PDAC) is one of the most malignant tumors because of its high metastatic ability. The glutamine (Gln)-deficient microenvironment contributes to PDAC metastasis; however, the underlying molecular mechanisms remain unclear. Here, we demonstrated that melanophilin (MLPH) promotes PDAC metastasis by inducing the regrowth of primary cilia. Using RNA sequencing, we found that MLPH was upregulated in Gln-deficient conditions. MLPH facilitated PDAC metastasis in vitro and in vivo. Clinically, high MLPH expression is positively correlated with metastasis and poor PDAC prognosis. MLPH localized to the centrosome and facilitated the regrowth of primary cilia. The primary ciliogenesis upregulated phospholipase C γ-1 (PLCG1) to promote PDAC metastasis. Interestingly, PLCG1 was localized to the primary cilia, and depletion of PLCG1 alleviated primary ciliogenesis, suggesting a feedforward role for PLCG1 in mediating primary ciliogenesis. Thus, our study revealed a novel function of the MLPH-primary cilia-PLCG1 axis in facilitating PDAC metastasis under Gln deficiency both in vitro and in vivo.

## Introduction

Pancreatic cancer is the eighth leading cause of cancer-related deaths worldwide, with a 5-year survival rate of approximately 9% [1]. The most common kind of pancreatic cancer (more than 95%) is pancreatic ductal adenocarcinoma (PDAC). PDAC arises through multistage genetic and histological progression from microscopic precursor lesions designated as pancreatic intraepithelial neoplasia (PanIN), which develop and progress over several years. Genetically, *KRAS* mutations initiate the development of pancreatic cancer, and more than 90% of PanINs harbor *KRAS* mutations. Mutations in *TP53*, *CDKN2A*, and *SMAD4* have also been associated with PDAC progression [2]. PDAC is a malignant tumor with a poor clinical outcome due to late diagnosis, resistance to chemotherapy, and metastasis [3]. PDAC metastasis remains one of the most challenging issues in pancreatic cancer therapy, and its underlying mechanisms are largely unknown.

PDAC is one of the most metastatic cancers owing to its desmoplastic microenvironment [4, 5]. Desmoplastic lesions typically contain several stromal cell types and abundant extracellular matrix but lack vascularization, thus causing persistent and severe hypoxia and nutrient deprivation [6]. Glutamine (Gln) is one of the most depleted nutrients in the PDAC desmoplastic microenvironment [7, 8]. Gln is the major carbon and nitrogen source for PDAC growth; therefore, PDAC cells are susceptible to Gln deficiency [9]. Short-term Gln deprivation for three days has been shown to promote PDAC metastasis by inducing epithelial-mesenchymal transition (EMT) [7]. However, the PDAC cells did not proliferate. In clinical PDAC samples, either the growth or metastasis of PDAC is increased in desmoplastic microenvironments. Thus, short-term Gln deprivation may not be suitable for deciphering how Gln deficiency induces PDAC metastasis.

The primary cilium, which appears in almost all cell types in the human body, is an immotile microtubule-based protrusion of the plasma membrane [10]. It is the cellular antenna that senses environmental signaling molecules for proper development and differentiation [11]. Primary cilia protrude from the distal end of the mother centriole. The attachment of Golgi-derived ciliary vesicles to the distal end of the mother centriole facilitates primary ciliogenesis [12]. After docking, the mother centriole protrudes into the vesicle and forms an axoneme [13]. This process is regulated by intraflagellar transporters (IFTs), which are bi-directional axoneme trafficking systems driven by microtubule motors. Extension of the axoneme is accompanied by the fusion of the ciliary sheath with the plasma membrane, followed by exposure of the ciliary membrane. Tubulins of the axoneme undergo several posttranslational modifications, such as acetylation, to maintain axoneme rigidity [14].

Defective primary ciliogenesis leads to tumorigenesis. During the progression of pancreatic cancer, primary cilia are gradually reduced during the transition from normal to PanIN and then to PDAC [15]. Therefore, the PDAC cells are devoid of primary cilia. This phenotype is also supported by mouse model studies. Loss of primary cilia by depletion of ciliary components, such as *Ift88* and *Kif3a*, in the pancreatic epithelium causes pancreatic ductal metaplasia [16]. The loss of primary cilia correlates with increased malignancy in several cancers, including prostate, ovarian, and other cancer cell types [17]. However, primary cilia may have tumor-promoting effects in medulloblastoma and basal cell carcinoma [18], suggesting that primary cilia may have varying effects on tumor progression in a tissue-specific manner. Interestingly, although PDAC cells do not grow primary cilia, our recent study showed that a subpopulation of PDAC cells regained the ability to grow primary cilia and that these ciliated PDAC cells were resistant to chemotherapy [19]. Clinical observations have shown that primary cilia can be detected in PDAC cells of patients with lymph node metastases [20]. Thus, at least in PDAC, the regrowth of primary cilia may lead to an even worse prognosis, such as chemoresistance in pancreatic cancers.

Melanophilin (MLPH) is mainly expressed in melanocytes and has been identified as a mutated gene in leaden mice [21]. MLPH transports melanosomes by moving them on actin filaments or microtubule networks [22, 23]. Since MLPH plays a vital role in mediating melanosome transportation, a deficiency in MLPH function impedes the transfer of melanosomes from melanocytes to keratinocytes, thereby causing deficient pigmentation in mammals [21]. In addition to its canonical role in protecting the skin against UV irradiation, recent studies have shown that MLPH participates in tumorigenesis. MLPH is aberrantly upregulated in radiation-resistant glioblastomas [24]. During the progression of glioblastoma radioresistance, MLPH is upregulated via O-GlcNAcylation, which inhibits its ubiquitination and proteasomal degradation. In addition, the overexpression of MLPH accelerates prostate cancer cell growth and malignancy [25]. Moreover, MLPH levels are positively correlated with advanced tumor status, resistance to chemoradiation, and poor survival in rectal cancers [26]. Collectively, MLPH overexpression is strongly associated with tumorigenesis and poor cancer outcomes; however, the link between MLPH and cancer metastasis remains unclear.

In this study, we found that MLPH was upregulated in Gln deficient conditions and that high MLPH expression facilitated PDAC metastasis both in vitro and in vivo. Notably, the regrowth of primary cilia played an important role in MLPH-mediated PDAC metastasis. Furthermore, primary cilia upregulated phospholipase C γ-1 (PLCG1) expression, thereby promoting EMT and invasion, and feedforward maintained primary ciliogenesis. Thus, our study not only demonstrates the role of primary cilia regrowth in driving PDAC metastasis but also elucidates a novel molecular mechanism by which Gln deficiency promotes PDAC metastasis.

## Results

### Glutamine deprivation promotes PDAC EMT and metastasis

Gln-deficient desmoplastic microenvironments contribute to PDAC metastasis; however, the underlying molecular mechanisms remain unclear. A recent study showed that short-term Gln deprivation for three days inhibited PDAC cell growth but promoted metastasis [7]. However, in clinical pathological samples, either the growth or metastasis of PDAC is increased in desmoplastic microenvironments, suggesting that PDAC adapts to long-term Gln-deficient microenvironments, gaining the ability to grow and metastasize [27]. To investigate the effects of Gln deprivation on PDAC metastasis, we cultured human PANC-1 cells and mouse KPC cells (PDAC cells derived from the KPC (Kras^G12D/+^; Trp53^R172H/+^) mouse model) in a Gln-deficient culture medium for three days (-Q, short-term) or three months (-QQ, long-term), followed by checking cell growth. The growth of -Q PANC-1 cells was dramatically inhibited; however, after long-term Gln deprivation, the growth of -QQ cells was similar to that of the parental cells in PANC-1 (Fig. S1A) and KPC cells (Fig. S1B). To further confirm our observations, the ability of cells to enter S phase was examined using an EdU incorporation assay. The proportion of EdU-positive cells increased in both parental and -QQ cells, but not in -Q cells (Fig. S1C–E). These data suggest that short-term Gln deprivation inhibits cell growth, but cells regain the ability to proliferate upon long-term Gln deficiency. A recent study showed that glutamate ammonia ligase (GLUL) expression is upregulated to promote PDAC cell growth upon Gln deficiency in samples from patients with pancreatic cancer and in mouse models [28]. In addition, we observed that -QQ PANC-1 cells underwent cell proliferation. Therefore, we examined the expression of GLUL. GLUL mRNA levels in -Q PANC-1 cells were similar to those in the parental cells; however, GLUL was dramatically upregulated in -QQ PANC-1 cells, suggesting that metabolic changes in -QQ cells promoted PDAC cell growth under Gln deficiency (Fig. S1F). Previous studies have shown that ERK activation and ATF4 upregulation facilitate PDAC EMT and invasion in -Q PANC-1 cells [7], so we checked whether these events also occurred in -QQ PANC-1 cells. ERK phosphorylation increased in -Q cells but not in -QQ cells (Fig. S1G). In addition, ATF4 was upregulated in -Q cells but was downregulated in -QQ cells (Fig. S1H). Unlike in -Q cells, these data suggest that distinct signaling pathways are activated in -QQ PANC-1 cells. Gln deficiency in tumor progression is a long-term outcome, and PDAC cells proliferate within these microenvironments. We thus used -QQ PANC-1 and KPC cells in subsequent experiments.

**Fig. 1:**
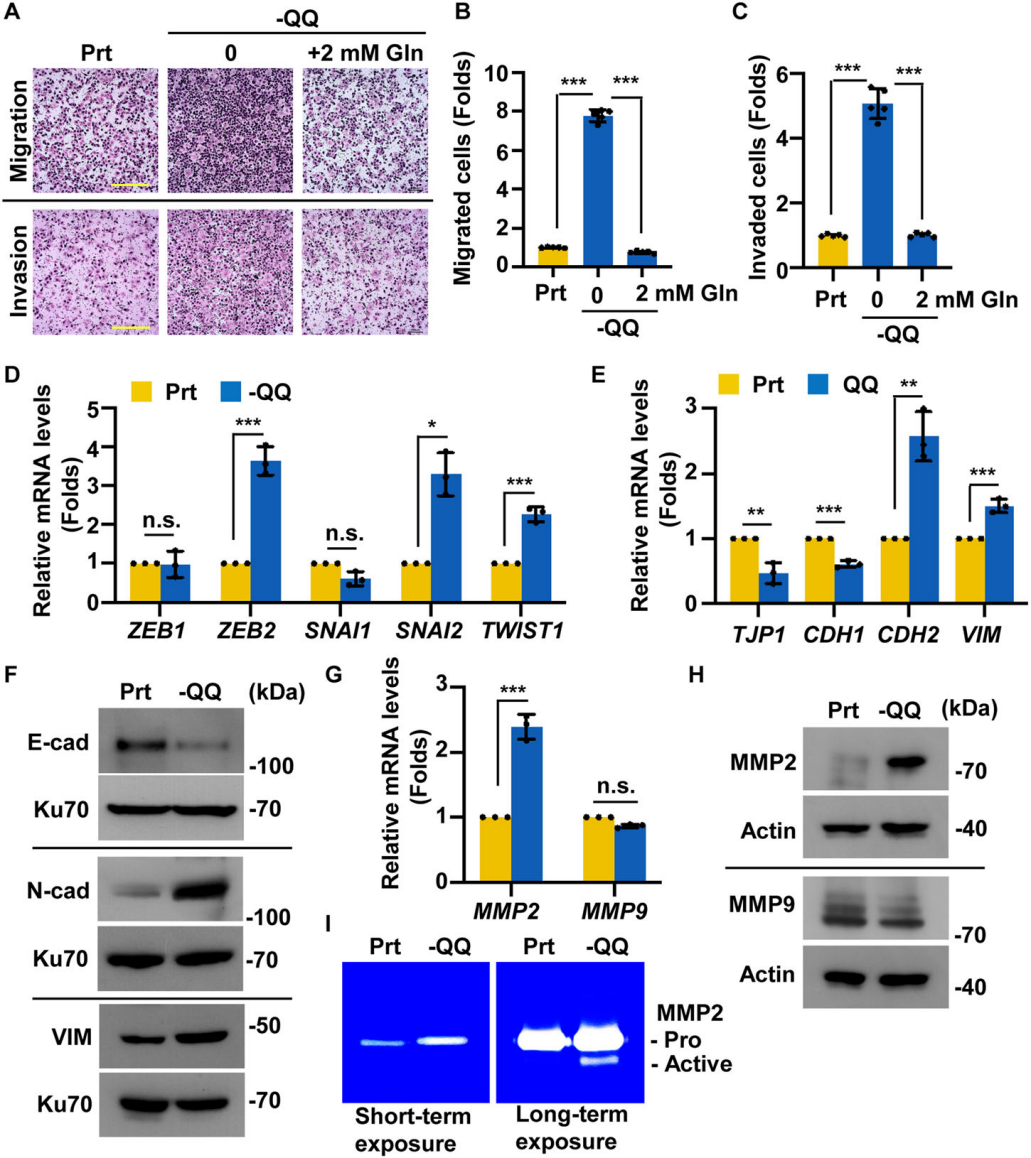
Glutamine deprivation promotes EMT and invasion in PANC-1 cells. **A**–**C** Gln-deficient condition promoted PANC-1 migration and invasion, and supplementation of Gln restored these phenotypes. **A** The cell invasion and migration ability of parental (Prt) cells and -QQ PANC-1 cells supplemented with or without two mM Gln were evaluated using Trans-well analysis. Scale bar, 200 μm. **B** Quantitative results of the relative migrated cell numbers were measured in (**A**). **C** Quantitative results of the relative invaded cell numbers were measured in (**A**). **D**–**F** Gln deficiency induced EMT in PANC-1 cells. **D** EMT-related transcription factors were upregulated in -QQ cells. Quantitative results of relative mRNA levels of *ZEB1*, *ZEB2*, *SNAI1*, *SNAI2*, and *TWIST1* in Prt or -QQ PANC-1 cells. **E** Epithelial markers were downregulated, and mesenchymal markers were upregulated in -QQ cells. Quantitative results of relative mRNA levels of ZO-1, E-cadherin (E-cad, *CDH1*), N-cadherin (N-cad, *CDH2*), and vimentin (VIM) in Prt or -QQ PANC-1 cells. **F** E-cadherin (E-cad), N-cadherin (N-cad), and vimentin (VIM) were upregulated -QQ cells. Extracts of Prt or -QQ PANC-1 cells were analyzed by western blot assay with antibodies against E-cad, N-cad, VIM, and Ku70 (loading control). **G**–**I** Upregulated MMP2 was observed in -QQ cells. **G** MMP2 but not MMP9 mRNA levels increased in -QQ PANC-1 cells. Quantitative results of relative mRNA levels of MMP2 and MMP9 in the Prt or -QQ PANC-1 cells. **H** MMP2 protein levels increased in -QQ cells. Extracts of Prt or -QQ PANC-1 cells were analyzed with western blot assay with antibodies against MMP2, MMP9, and actin. **I** MMP2 activity increased in -QQ cells. MMP activities were analyzed with gelatin zymography in Prt and -QQ PANC-1 cells. Pro: pro-MMP2, active: activated MMP2. Data are represented as the mean ± SD of three independent experiments. n.s. no significance, *P < 0.05, **P < 0.01, ***P < 0.001.

Cell metastasis is characterized by increased cell migration and invasion. The migratory ability of -QQ PANC-1 and KPC cells increased, and re-supplementation with Gln alleviated Gln deficiency-induced migration (Fig. 1A upper panel, 1B, and S2A). Gln deficiency promoted cell invasion, and Gln supplementation rescued this phenotype (Fig. 1A lower panel, 1C, and S2B). These data suggest that Gln deficiency promotes PDAC cell migration and invasion.

EMT was next examined. The mRNA level of EMT-activating transcription factors (EMT-TF), including zinc finger E-box binding homeobox 2 (*ZEB2*), snail family transcriptional repressor 2 (*SNAI2*), and Twist family BHLH transcription factor 1 (*TWIST1*), were upregulated in -QQ cells (Fig. 1D). The epithelial markers ZO-1 (also known as tight junction protein 1, *TJP1*) and E-cadherin (*CDH1*) were downregulated, whereas the mesenchymal markers N-cadherin (*CDH2*) and vimentin (*VIM*) were upregulated in -QQ cells (Fig. 1E). These data were further supported by examining EMT markers by western blotting (Fig. 1F and S2C) and immunofluorescence staining (Fig. S2D) in PANC-1 and KPC cells. In addition, the EMT of -QQ cells was restored when the cells were re-supplemented with Gln (Fig. S2E), suggesting that Gln deficiency promoted PDAC EMT. Matrix metalloproteinases (MMPs) contribute to cancer metastasis, and their expression and activity were examined. The expression of MMP2, but not of MMP9, increased significantly at the mRNA and protein levels in -QQ cells (Fig. 1G, H). In addition, the MMP2 activity was upregulated in -QQ cells as shown by gelatin zymographic assay (Fig. 1I), suggesting that Gln deficiency upregulated and activated MMP2. Collectively, these data suggest that Gln deficiency promotes the EMT and invasion of PDAC cells.

### MLPH upregulation promotes PDAC metastasis under Gln deficiency

**Fig. 2:**
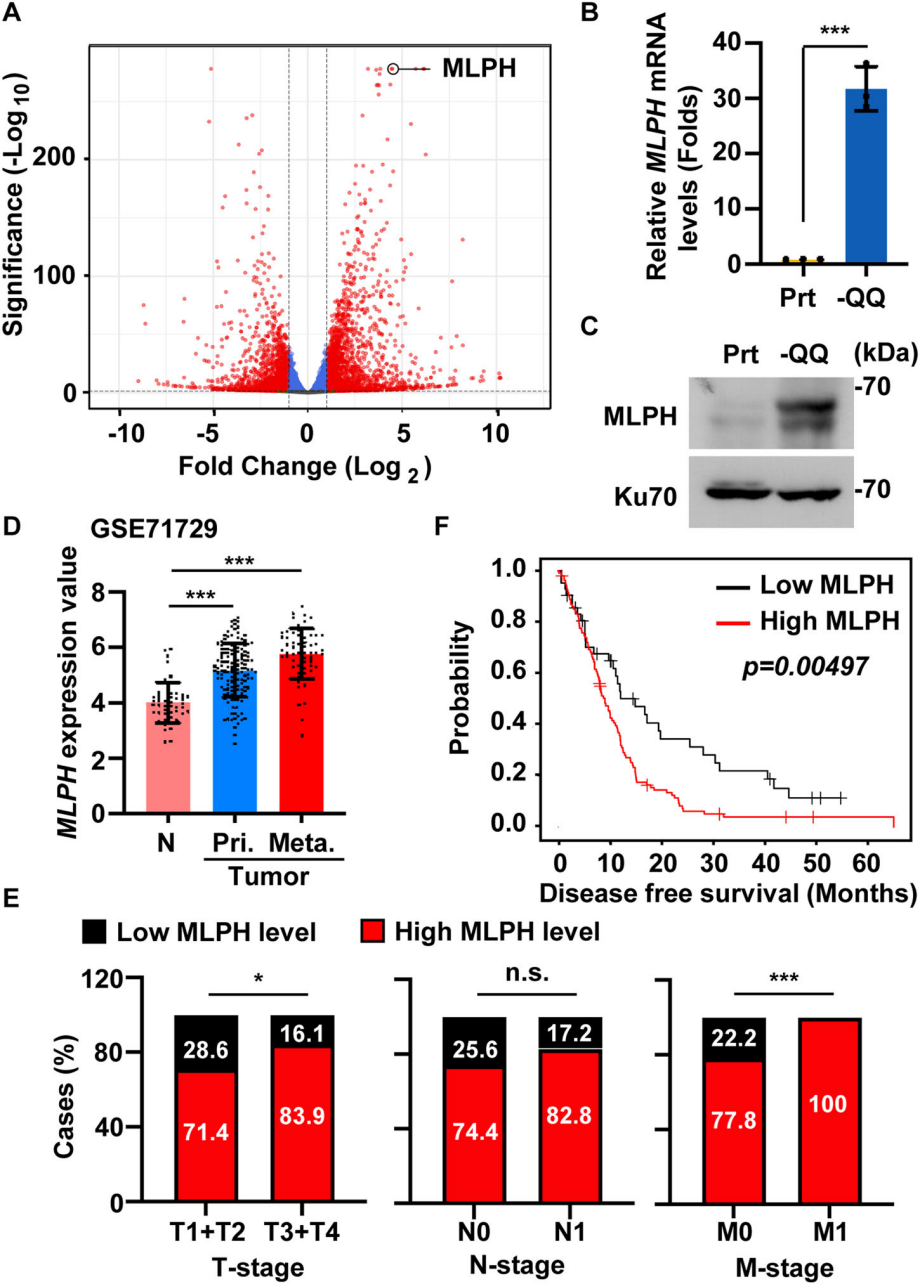
MLPH levels were positively correlated with PDAC tumorigenesis and metastasis. **A**–**C** MLPH was upregulated in -QQ PANC-1 cells. **A** Differentially expressed genes in parental (Prt) or -QQ PANC-1 cells were identified by the Bulk RNA sequence analysis shown in the Volcano Plot. Red dots represent genes with significant differences, and blue dots represent genes without significant differences. **B** MLPH mRNA levels increased in -QQ cells. Quantitative results of relative mRNA levels of MLPH in Prt or -QQ PANC-1 cells. **C** MLPH protein levels increased in -QQ cells. Extracts of Prt and -QQ PANC-1 cells were analyzed by western blot assay with antibodies against MLPH and Ku70 (loading control). **D** MLPH was upregulated in metastatic PDAC tissues compared to benign pancreas tissue in PDAC patients. The mRNA gene expression of MLPH in normal (N), primary tumor (Pri.), and metastatic tumor (Meta.) were obtained from the GEO database: GEO71729. **E** PDAC patients with higher MLPH expression showed poorer prognosis. Data analyzed from NCKUH cohort. **F** High MLPH expression positively correlated with poor disease-free survival. Disease-free survival was analyzed using the Kaplan–Meier method. Data were analyzed from The Cancer Genome Atlas (TCGA) database. *P* values were determined using the log-rank test. Data are represented as the mean ± SD of three independent experiments. n.s. no significance, *P < 0.05, ***P < 0.001.

Bulk RNA sequence analysis was performed to investigate the molecular mechanism by which Gln deficiency contributes to PDAC invasion. Several genes were identified, and MLPH was one of the most highly upregulated genes in -QQ PANC-1 cells (Fig. 2A). Here, we are interested in MLPH as the overexpression of MLPH has been associated with poorer patient survival and increased metastasis in prostate and rectal cancers [26, 29]. The expressions of MLPH were further confirmed by quantitative RT-PCR and western blotting in PANC-1 cells and KPC cells (Fig. 2B, C and S3A), supporting the hypothesis that MLPH is upregulated in -QQ cells. The clinical significance of MLPH in PDAC was then examined. Independent microarray datasets obtained from the Gene Expression Omnibus (GEO) database (GSE15471, GSE28735, GSE62452, and GSE71729) were analyzed. The results showed that MLPH levels were higher in PDAC tumor tissues than in normal pancreatic tissues in all datasets (Fig. 2D, S3B). Notably, analysis of clinical samples from the National Cheng Kung University Hospital (NCKUH) using the Tumor-Node-Metastasis (TNM) staging system revealed that patients with PDAC with higher MLPH expression showed poorer prognosis (Fig. 2E). These results were further confirmed using the GEO database, which showed that MLPH expression was higher in metastatic tumors than in primary tumors (Fig. 2D). Importantly, high MLPH expression positively correlated with poor disease-free survival (DFS) and overall survival (OS) in patients with PDAC from both The Cancer Genome Atlas (TCGA) (Fig. 2F) and NCKUH (Fig. S3C). Taken together, MLPH levels were positively correlated with PDAC tumorigenesis and metastasis.

**Fig. 3:**
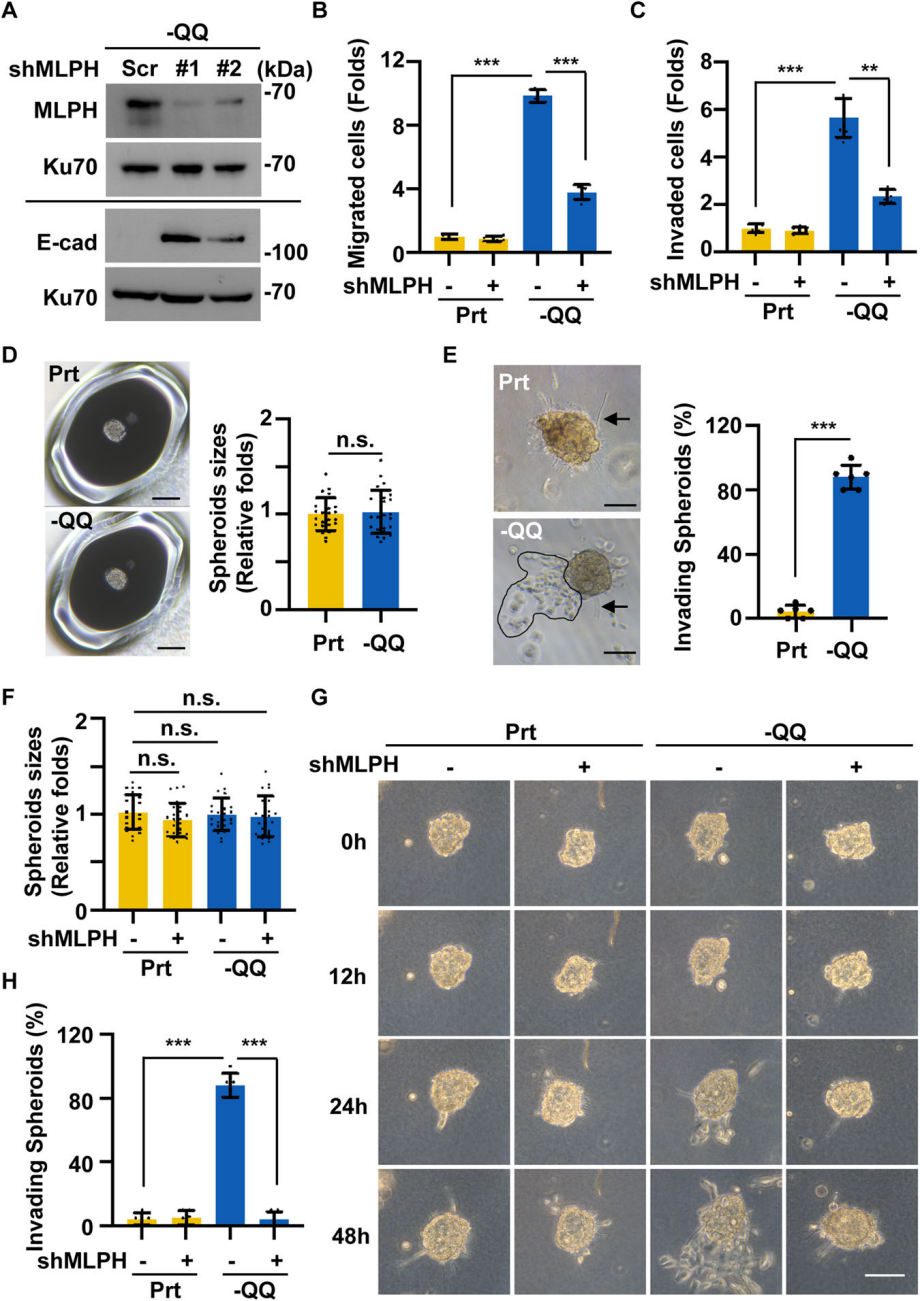
MLPH upregulation promotes PDAC invasion in the 3D tumor spheroids. **A** MLPH depletion restored E-cadherin expression. MLPH was depleted efficiently. Extracts of -QQ PANC-1 cells infected with lentivirus containing shRNA against scramble control depletion (shScr), or MLPH (#1 and #2) were analyzed by western blot assay with antibodies against MLPH, E-cadherin (E-cad), or Ku70 (loading control). MLPH promoted PDAC migration and invasion. Quantitative results of (**B**) migrated or (**C**) invaded parental (Prt) or -QQ cells in the absence or presence of shRNA against MLPH (shMLPH#1). **D** Tumor spheroid sizes in Prt or -QQ PANC-1 groups showed no difference. Scale bar, 100 µm. **E** Invaded cells were observed in the -QQ tumor spheroids. -QQ spheroids showed invaded cells that escaped from the spheroids (dashed circle). Scale bar, 50 µm. Multiple protrusions were observed in both Prt and -QQ PANC-1 spheroids (arrow). **F** Depletion of MLPH did not affect tumor spheroid sizes. Quantitative results of Prt or -QQ 3D tumor spheroids in the absence or presence of shRNA against MLPH (shMLPH#1). **G**, **H** Invading spheroids increased in a time-dependent manner. Observation of invaded cells in scramble control or MLPH-deficient Prt or -QQ spheroids. Scale bar, 50 µm. **H** Quantitative results of (**G**). Data are represented as the mean ± SD of three independent experiments. n.s. no significance, **P < 0.01, ***P < 0.001.

To confirm that MLPH participates in PDAC metastasis, MLPH was depleted by infecting cells with lentiviruses containing different short hairpin RNA (shRNA) sequences against MLPH (shMLPH#1 and shMLPH#2), and the EMT and metastatic ability were examined. MLPH was efficiently depleted by both shMLPH#1 and shMLPH#2, and E-cadherin expression was restored in MLPH-deficient cells (Fig. 3A). In addition, MLPH depletion alleviated Gln deficiency-induced migration and invasion (Fig. 3B, C), suggesting that MLPH participates in PDAC EMT and invasion under conditions of Gln deficiency. In addition to Gln deficiency, hypoxia is an important hallmark and contributes to metastasis in the PDAC tumor microenvironment [30]. We then tested whether hypoxia promoted PDAC invasion via MLPH. Both Gln deprivation and hypoxia promoted PDAC invasion (Fig. S3D). However, MLPH was upregulated in Gln deprivation but not under hypoxia conditions (Fig. S3E). Thus, our data suggest that either hypoxia or glutamine deficiency promotes PDAC metastasis; however, MLPH is upregulated by Gln deficiency for PDAC metastasis. Taken together, we found that Gln deficiency promotes metastasis through the upregulation of MLPH.

Next, we established three-dimensional (3D) PDAC tumor spheroids using a multicellular tumor spheroid (MCTS) system to support our observations further. Both parental and -QQ PANC-1 cells formed PDAC tumor spheroids of similar sizes in the MCTS system (Fig. 3D). The metastatic ability of PDAC tumor spheroids was examined by embedding PDAC tumor spheroids within collagen-based scaffolds. Several protrusions were observed in both parental and -QQ tumor spheroids 24 h after embedding (Fig. 3E, arrows). In addition, some cells disseminated from the spheroids and invaded the matrixes in -QQ spheroids (Fig. 3E, circle). Spheroids with disseminated cells were defined as invading spheroids. The proportions of invading spheroids were increased in -QQ groups when compared with parental groups (Fig. 3E). Next, we examined whether MLPH affected the cell dissemination of the 3D tumor spheroids. MLPH depletion did not affect the size of the 3D tumor spheroids (Fig. 3F); however, the proportions of invading spheroids were increased in -QQ groups, and depletion of MLPH reduced the phenotype (Fig. 3G, H). These data suggested that MLPH promoted PDAC metastasis in in vitro cell cultures and 3D PDAC tumor spheroids.

**Fig. 4:**
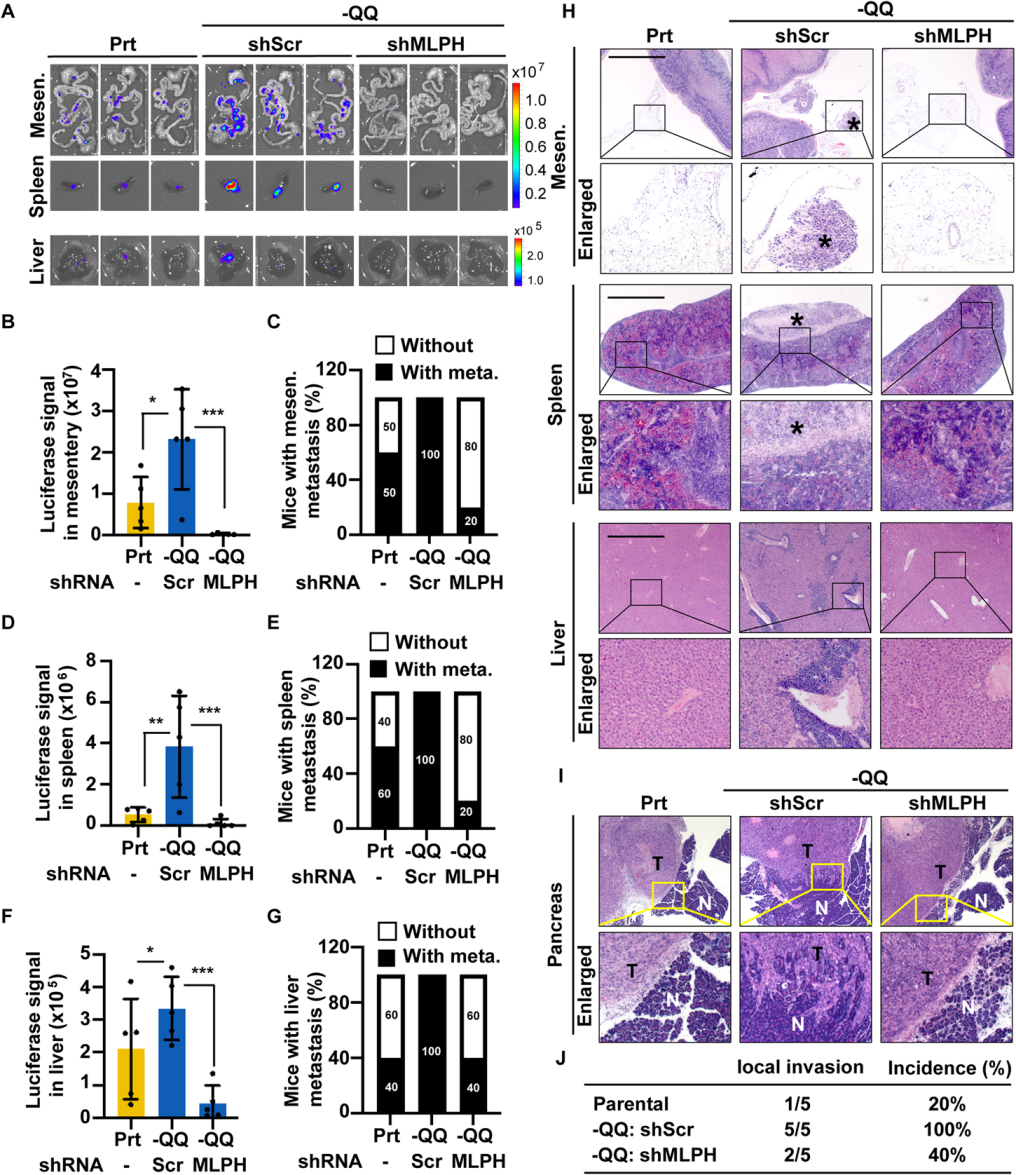
MLPH upregulation promotes PDAC metastasis under Gln deficiency in the orthotopic mouse model. **A** Representative IVIS images were shown in the mesentery (Mesen.), spleen, and liver of Prt, scramble control depletion -QQ (-QQ-shScr), and MLPH depleted-QQ (-QQ-shMLPH) groups. Quantitative relative luciferase signals in (**B**) mesentery, (**D**) spleen, and (**F**) liver, and percentages of metastasis of (**C**) mesentery (Mesen.), (**E**) spleen, and (**G**) liver. **H** Representative H&E staining of the mesentery (Mesen.), spleen, and liver of Prt, -QQ-shScr, and -QQ-shMLPH groups. Metastatic colonies were labeled as asterisks. Scale bar, 100 μm. **I**–**J** MLPH promoted pancreatic local invasion. **I** Representative H&E staining of the pancreas of Prt, -QQ-shScr, and -QQ-shMLPH groups. T: tumor tissues, N: adjacent normal tissues. Scale bar, 100 μm. **J** Quantitative results of pancreatic local invasion were calculated in Prt, -QQ-shScr, and -QQ-shMLPH groups. Data are represented as the mean ± SD of three independent experiments. n.s. no significance, *P < 0.05, **P < 0.01, ***P < 0.001.

To further confirm these findings, we examined whether MLPH promoted PDAC metastasis in vivo using an orthotopic xenograft mouse model. First, we generated stable luciferase-expressing PANC-1 parental, -QQ, and MLPH-deficient -QQ cells to trace PDAC metastasis using in vivo bioluminescence imaging systems (IVIS). We obtained stable luciferase-expressing -QQ PANC-1 cells by culturing the cells in a Gln-deficient medium for three months. MLPH was upregulated, whereas E-cadherin was downregulated, and the depletion of MLPH rescued the expression of E-cadherin in stable luciferase-expressing -QQ cells (Fig. S4A–C). In addition, the migratory and invasive abilities were increased in stable luciferase-expressing -QQ cells (Fig. S4D, E). Stable luciferase-expressing parental (Prt), scramble control -QQ (-QQ-shScr), and MLPH-deficient -QQ (-QQ-shMLPH) PANC-1 cells were orthotopically inoculated into the pancreas of non obese diabetic/severe combined immunodeficiency NOD/SCID mice. Seven weeks after inoculation, body weights and primary tumor volumes showed no significant difference between parental, -QQ-shScr, and -QQ-shMLPH groups (Fig. S4F–H). Interestingly, by examining the luciferase signals, we observed that metastatic colonies in the mesentery (Fig. 4A–C), spleen (Fig. 4A, D, E), and liver (Fig. 4A, F, G) were increased in the -QQ-shScr group but were reduced dramatically in the -QQ-shMLPH group. These data were further supported by the observation of metastatic colonies with hematoxylin and eosin (H&E) staining (Fig. 4H). In addition to metastasis in the mesentery, spleen, and liver, pancreatic local invasion was also observed in the -QQ-shScr group but was reduced in the -QQ-shMLPH group (Fig. 4I–J). Next, to test the immunocompetent effect on PDAC metastasis, the parental (Prt), scramble control -QQ (-QQ-shScr), and MLPH-deficient -QQ (-QQ-shMLPH) mouse KPC cells were orthotopically inoculated into the pancreas of C57BL/6 J mice. Seven weeks after inoculation, body weights and primary tumor volumes showed no significant difference between parental, -QQ-shScr, and -QQ-shMLPH groups (Fig. S5A–C). Interestingly, we observed that metastatic colonies in the mesentery, spleen, and liver were increased in the -QQ-shScr group but were reduced dramatically in the -QQ-shMLPH group (Fig. S5D, E). These data were further supported by the observation of metastatic colonies with hematoxylin and eosin (H&E) staining (Fig. S5F). In addition to metastasis in the mesentery, spleen, and liver, pancreatic local invasion was also observed in the -QQ-shScr group but was reduced in the -QQ-shMLPH group (Fig. S5G, H). Taken together, the data show that MLPH promoted PDAC metastasis under Gln deficiency both in vitro and in vivo.

### Primary cilia promote EMT and metastasis upon Gln deficiency

**Fig. 5:**
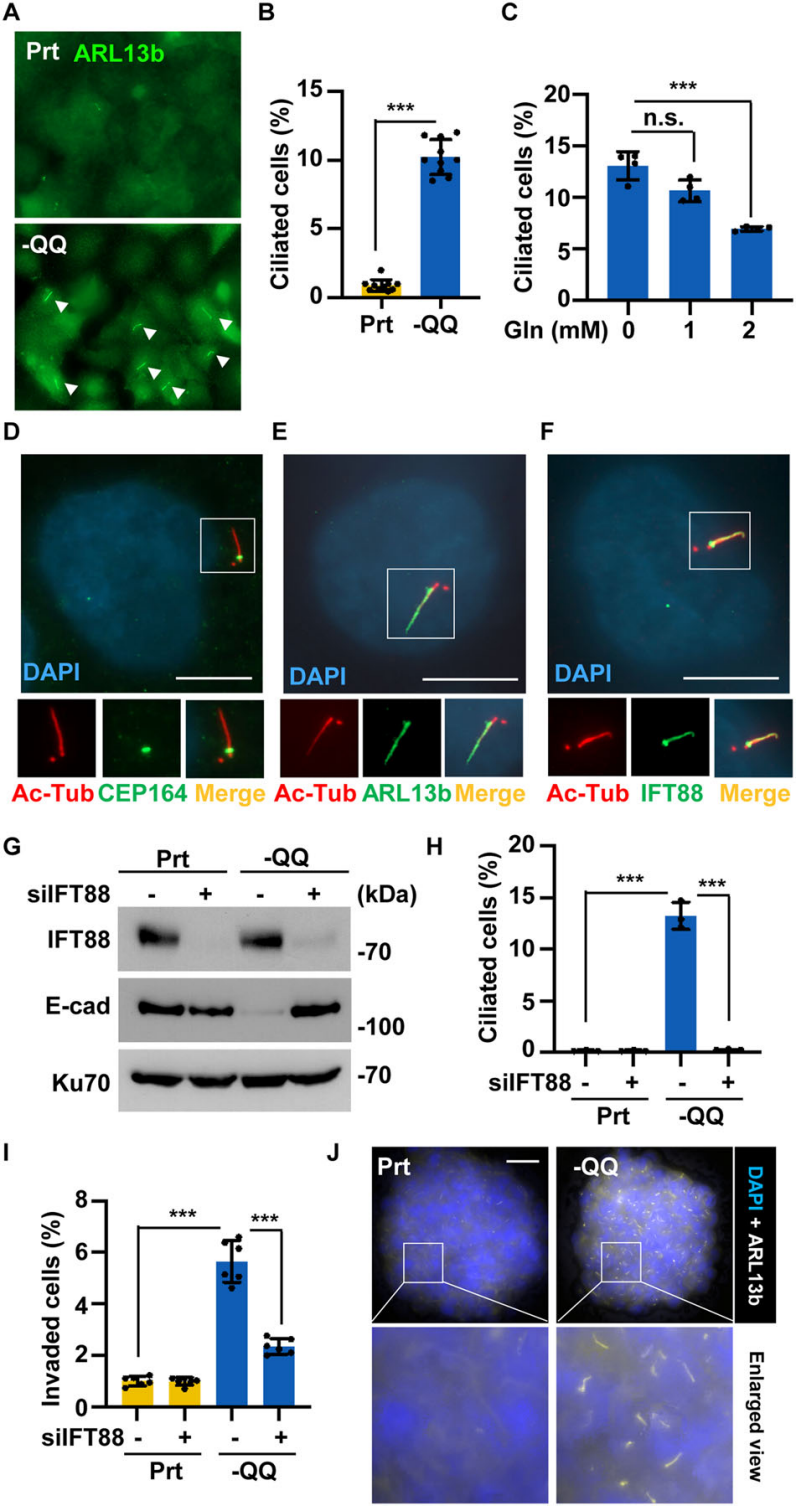
Primary cilia promote EMT and metastasis upon Gln deficiency. **A, B** Primary cilia formation was observed in -QQ PANC-1 cells. (A) Primary cilia were detected by immunofluorescence staining with an antibody against ARL13b (green) in Prt or -QQ PANC-1 cells. DNA was stained with DAPI (blue). Scale bar, 20 µm. **B** Quantitative results of the frequency of ciliated cells in (**A**). **C** Gln supplementation inhibited primary ciliogenesis in -QQ cells. Quantitative results of the frequency of ciliated cells supplemented with different dosages of Gln in -QQ PANC-1 cells. Ciliary components were detected by immunofluorescence staining with antibodies against acetylated tubulin (Ac-tub, **D**–**F**), CEP164 (**D**), ARL13b (**E**), and IFT88 (**F**). DNA was stained with DAPI. Scale bar, 10 µm. **G**–**I** Primary cilia contributed to EMT and cell invasion. (G) IFT88 was depleted efficiently. Extracts of parental (Prt) or -QQ PANC-1 cells in the absence or presence of siRNA against IFT88 (siIFT88) were analyzed by western blot assay with antibodies against IFT88, E-cadherin (E-cad), or Ku70. (H) Quantitative results of the frequency of ciliated cells in Prt or -QQ PANC-1 cells in the absence or presence of siRNA against IFT88 (siIFT88). **I** Quantitative results of the relative invaded cells in Prt or -QQ PANC-1 cells in the presence or absence of siRNA against IFT88 (siIFT88). **J** The frequency and length of primary cilia were significantly increased in the -QQ PDAC spheroids. Primary cilia were detected by immunofluorescence staining with an antibody against ARL13b (white) in Prt or -QQ PANC-1 spheroids. DNA was stained with DAPI (blue). Scale bar, 10 µm. Data are represented as the mean ± SD of three independent experiments. n.s. no significance, ***P < 0.001.

PDAC cells lack primary cilia; however, our recent study showed that the regrowth of primary cilia contributed to PDAC chemoresistance [19]. Additionally, primary cilia promoted trophoblast invasion during early implantation [31]. Thus, we speculated that primary cilia facilitate PDAC metastasis under Gln deprivation. Using immunofluorescence staining, we found that primary cilia were present in -QQ PANC-1 and -QQ KPC cells (Fig. 5A, B and S6A, B), and that Gln supplementation alleviated primary ciliogenesis in a dose-dependent manner (Fig. 5C). Next, the genes related to primary ciliogenesis were examined. The mRNA levels of *IFT43* and *IFT88* were upregulated in -QQ PANC-1 cells (Fig. S6C, D). Thus, Gln deficiency facilitates primary ciliogenesis. The structures of the primary cilia were examined. Axoneme (acetylated-tubulin), distal appendages of mother centriole (CEP164), ciliary membrane (ARL13b), and intraflagellar transporter (IFT88) were detected in the primary cilia of -QQ PANC-1 cells, suggesting these primary cilia contained essential ciliary components (Fig. 5D–F).

We examined whether primary cilia contribute to PDAC EMT and invasion. Primary cilia were disrupted by transfecting the cells with small interfering RNA (siRNA) against the ciliary gene *IFT88. IFT88* was efficiently depleted, and primary ciliogenesis was inhibited in IFT88-deficient cells (Fig. 5G, H). Gln deficiency induced EMT, migration, and invasion; however, these phenotypes were rescued when the primary cilia were disrupted (Figs. 5G, I, and S6E). To confirm that primary cilia contribute to PDAC invasion, another ciliary gene, *CEP164*, was depleted. CEP164 depletion inhibited primary ciliogenesis (Fig. S6F, G) and alleviated Gln deficiency-induced EMT, migration, and invasion (Fig. S6H-J). These data suggested that primary cilia participate in PDAC EMT and invasion under Gln-deficient conditions.

Next, the role of primary ciliogenesis in promoting PDAC invasion was confirmed using 3D tumor spheroid cultures. To establish tumor spheroids, we infected cells with lentiviruses containing shRNA against IFT88 (five sequences, #1 to #5). The shIFT88#1 efficiently suppressed IFT88 expression (Fig. S7A). IFT88 depletion did not affect the size of the tumor spheroids (Fig. S7B). Compared to the parental PDAC spheroids, the frequency and length of primary cilia were significantly increased in the -QQ PDAC spheroids (Fig. 5J). Notably, invading spheroids increased in -QQ spheroids and were reduced in *IFT88*-deficient -QQ spheroids (Fig. S7C). Thus, our data suggest that primary cilia promote PDAC cell invasion under Gln deprivation.

The tumor microenvironment promotes PDAC metastasis and chemoresistance [32]. We then tested whether primary cilia promote chemoresistance under Gln deprivation. Compared to parental cells, -QQ PANC-1 cells were less sensitive to gemcitabine treatment. However, when primary cilia were depleted, the growth of -QQ PANC-1 cells was reduced (Fig. S6K). These data suggest that primary cilia protect PDAC cells against gemcitabine treatment under Gln deprivation.

### MLPH promotes primary cilia formation upon Gln deficiency

**Fig. 6:**
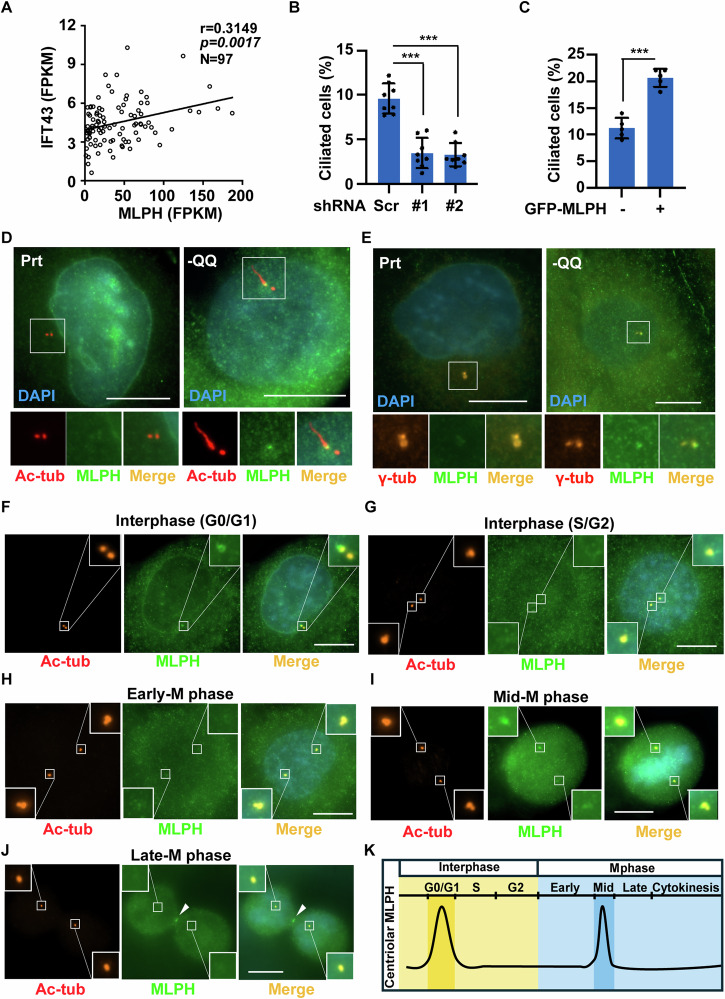
MLPH promotes primary cilia formation upon Gln deficiency. **A** IFT43 level positively correlated with MLPH expression. Scatter plot showed the relationship between genome-wide co-expression correlations for *IFT43* and *MLPH* in pancreatic cancer tissues of NCKUH. (**B**-**E**) MLPH regulated primary ciliogenesis. **B** Depletion of MLPH decreased primary cilia formation in -QQ PANC-1 cells. Quantitative results of frequency of ciliated cells in -QQ PANC-1 cells infected with lentivirus containing shRNA against scramble control depletion -QQ (Scr) and MLPH (#1 and #2). **C** Overexpression of MLPH induced primary ciliogenesis. Quantitative results of frequency of ciliated cells in -QQ cells transfected with GFP or GFP-tagged MLPH. **D** MLPH localized to the base of primary cilia. Double staining of Prt and -QQ PANC-1 cells with antibodies against acetylated tubulin (Ac-tub, red) and MLPH (green). DNA was stained with DAPI (blue). Scale bar, 10 µm. **E** MLPH localized to centrosome. Double staining of Prt and -QQ PANC-1 cells with antibodies against gamma-tubulin (γ-tub, orange) and MLPH (green). DNA was stained with DAPI (blue). Scale bar, 10 µm. The localization of MLPH on centrosome from (**F**–**G**) interphase to (**H**–**J**) mitosis. Double staining of -QQ PANC-1 cells with antibodies against MLPH (green) and acetylated tubulin (Ac-tub, red). DNA was stained with DAPI (blue). Scale bars, 10 μm. Insets are magnification of centrioles. **K** Schematic representation of the expression of centriolar MLPH from interphase to mitosis. Data are represented as the mean ± SD of three independent experiments. ***P < 0.001.

Gln deficiency upregulated MLPH and induced primary ciliogenesis. Therefore, we investigated whether MLPH contributed to primary ciliogenesis. By analyzing the clinical data, we found that MLPH had a significant positive correlation with the ciliary-related gene *IFT43* (Fig. 6A). In addition, depletion of MLPH blocked primary ciliogenesis (Fig. 6B), whereas overexpression of MLPH facilitated primary cilia formation (Fig. 6C, S8A). Examination of the subcellular localization of MLPH revealed that it was not only present in the cytoplasm but also localized at the base of primary cilia (Fig. 6D) and centrosome (Fig. 6E), implying that MLPH was recruited to the centrosome to promote primary cilia formation. After microtubule depolymerization under nocadazole treatment, MLPH is still localized on the centrosome, suggesting that MLPH is targeted to the centrosome independent of microtubule transport (Fig. S8B).

Next, we examined the centriolar targeting of MLPH. During the G0/G1 phase, MLPH was asymmetrically detected at one centriole, and this signal was reduced during the S/G2 phase. In the M phase, MLPH was only detected during mid-M phase. Thus, the expression of MLPH was highest during the G0/G1 phase and mid-mitosis (Fig. 6F–K). Collectively, these findings suggest that Gln deprivation upregulates MLPH, promoting primary cilia formation.

### Primary cilia upregulate phospholipase C gamma 1(PLCG1) to facilitate PDAC metastasis

**Fig. 7:**
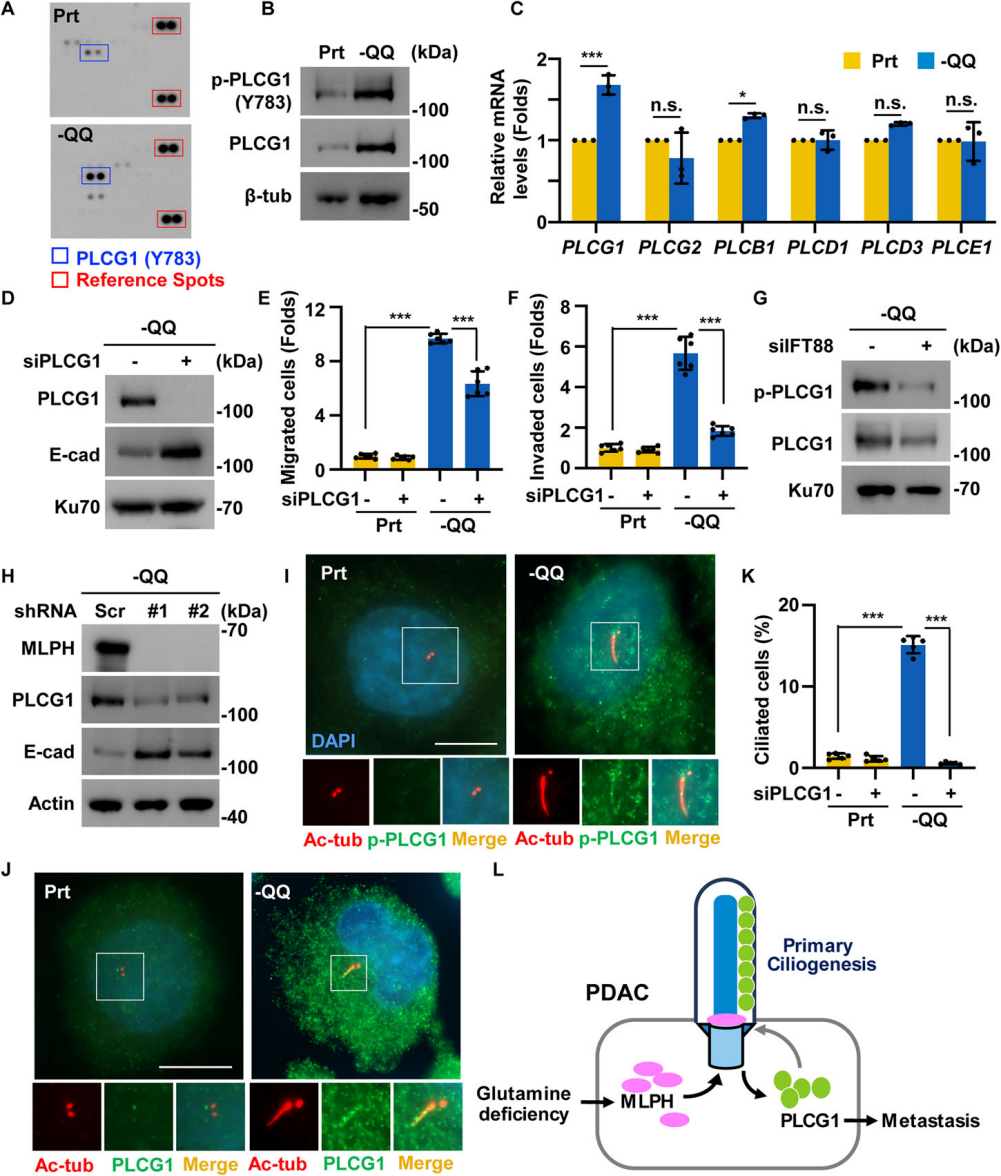
Primary cilia upregulate phospholipase C gamma 1(PLCG1) to facilitate PDAC metastasis. **A**, **B** PLCG1 was activated in -QQ PANC-1 cells. **A** Phosphorylated PLCG1 increased as shown by human phospho-kinase array analysis in Prt and -QQ PANC-1 cells. The red line shows reference spots, whereas the blue line indicates the phosphorylation of Y783 of PLCG1. **B** PLCG1 was phosphorylated at tyrosine 783 in -QQ cells. Extracts of parental (Prt) or -QQ PANC-1 cells were analyzed by western blot assay with antibodies against phosphorylated PLCG1 at Y783, PLCG1, and tubulin. **C** The PLCG1 mRNA level increased in -QQ cells. Quantitative results of relative phospholipase C family mRNA levels were analyzed by qPCR in Prt or -QQ PANC-1 cells. **D**–**F** PLCG1 promoted EMT, cell migration, and invasion. **D** Depletion of PLCG1 alleviated EMT in -QQ cells. Extracts of -QQ PANC-1 cells transfected with siRNA against PLCG1 (siPLCG1) were analyzed by western blot assay with antibodies against PLCG1, E-cadherin (E-cad), and Ku70. **E**, **F** Depletion of PLCG1 alleviated cell migration and invasion of -QQ cells. Quantitative results of the relative (**E**) migrated and (**F**) invaded cell numbers in Prt or -QQ PANC-1 cells in the presence or absence of siRNA against PLCG1 (siPLCG1). **G** Depletion of IFT88 decreased PLCG1 expression. Extracts of -QQ PANC-1 cells in the absence or presence of siRNA against IFT88 (siIFT88) were analyzed by western blot assay with antibodies against phosphorylated PLCG1 (Y783), PLCG1, and Ku70. **H** MLPH depletion decreased PLCG1 expression and restored EMT. Extracts of -QQ-shScr or -QQ-shMLPH#1 or #2 PANC-1 cells were analyzed by western blot assay with antibodies against MLPH, PLCG1, E-cadherin (E-cad), and actin. **I**–**J** PLCG1 and phosphorylated PLCG1 at Y783 (p-PLCG1) localized to the primary cilia. Immunofluorescence staining of Prt or -QQ PANC-1 cells with antibodies against acetylated tubulin (Ac-tub) and (**I**) p-PLCG1 and (**J**) PLCG1. DNA was stained with DAPI (blue). Scale bar, 10 µm. **K** Depletion of PLCG1 inhibited primary ciliogenesis. Quantitative results of the frequency of ciliated cells of Prt or -QQ PANC-1 cells were shown in the presence or absence of siRNA against PLCG1 (siPLCG1). **L** Graphic abstract. When PDAC cells suffered a Gln deprivation condition, MLPH was upregulated and recruited to the centrosome to facilitate primary ciliogenesis. The primary cilia induced and activated PLCG1 to promote EMT, invasion, and metastasis. Besides, PLCG1 was recruited to the primary cilia, thereby feedforward maintaining primary ciliogenesis. Data are represented as the mean ± SD of three independent experiments. n.s. no significance, *P < 0.05, ***P < 0.001.

MLPH induced primary cilia formation to promote PDAC metastasis. We next identified the underlying signaling pathway using a human phosphor-kinase array. Among several phosphorylated proteins, the phosphorylation of phospholipase C gamma 1 (PLCG1) at tyrosine 783 (Y783) increased dramatically in -QQ cells (Fig. 7A). These results were confirmed by western blot analysis. Phosphorylation at Y738 of PLCG1 was significantly increased in -QQ PANC-1 and -QQ KPC cells. Interestingly, non-phosphorylated PLCG1 levels were also elevated (Fig. 7B, S9A), suggesting that glutamine deficiency upregulates PLCG1 expression. Then, the mRNA levels of the PLC family genes were examined. Among these PLC family genes, PLCG1 was the most upregulated in -QQ PANC-1 cells (Fig. 7C). Collectively, Gln deficiency upregulates the expressions of PLCG1.

Next, we examined the association between PLCG1 expression and PDAC tumorigenesis. GEO database analysis (GSE15471, GSE28735, and GSE62452) showed that PLCG1 expression was higher in PDAC tissues (Fig. S9B), suggesting that PLCG1 expression positively correlated with PDAC tumorigenesis. We examined whether PLCG1 participates in EMT, migration, and invasion. PLCG1 depletion by transfecting cells with siRNAs against PLCG1 restored E-cadherin expression and inhibited PANC-1 cell migration and invasion (Fig. 7D–F). Blocking PLCG1 activation by treating cells with the selective inhibitor U-73122 rescued EMT, migration, and invasion of -QQ PANC-1 cells (Fig. S9C–E). Thus, PLCG1 promotes EMT, cell migration, and invasion during Gln deficiency.

We examined whether the expression of PLCG1 was regulated by primary ciliogenesis. Gln deficiency induced PLCG1 expression; however, depletion of the ciliary genes *IFT88* or *CEP164* alleviated PLCG1 upregulation (Fig. 7G, S9F), suggesting a role for primary cilia in promoting PLCG1 expression. MLPH induced primary cilia formation, and we examined whether MLPH contributed to PLCG1 upregulation. Depletion of MLPH with two specific shRNAs inhibited the expression of PLCG1 upon Gln deficiency (Fig. 7H). Thus, our data suggested that MLPH-regulated primary cilia contribute to PLCG1 upregulation under Gln-deficient conditions. The subcellular localization of PLCG1 was examined using immunofluorescence staining. Both phosphorylated-PLCG1 and PLCG1 were upregulated and localized to primary cilia (Fig. 7I–J), suggesting that active PLCG1 was recruited to primary cilia under Gln deficiency. Ciliary proteins maintain primary ciliogenesis, we thus tested whether PLCG1 plays a role in primary ciliogenesis. Depletion or inhibition of PLCG1 inhibited primary ciliogenesis in -QQ cells (Fig. 7K, S9G), suggesting that PLCG1 maintains primary cilia through positive feedforward regulation. Collectively, our data show that Gln deprivation-induced primary cilia promote PLCG1 expression and ciliary localization, and that active PLCG1 promotes PDAC metastasis and maintains primary ciliogenesis.

## Discussion

Gln-deficient microenvironments promote PDAC metastasis. However, the underlying molecular mechanisms remain unclear. Here, we show that Gln deficiency promotes PDAC EMT and metastasis by upregulating MLPH, both in vitro and in vivo. Mechanistically, MLPH is upregulated and recruited to the centrosomes to facilitate primary ciliogenesis. Primary cilia further induce and activate PLCG1 to promote EMT and invasion in PDAC. Active PLCG1 was recruited to primary cilia, and depletion or inhibition of PLCG1 disrupted primary cilia, suggesting that PLCG1 feedforward maintained primary ciliogenesis (Fig. 7L). Thus, our study not only uncovered how Gln deficiency promotes PDAC metastasis, but also deciphered the novel function of MLPH in mediating primary ciliogenesis.

MLPH is mainly expressed in melanocytes and regulates melanosome transport [21]. Our data showed that during Gln deficiency, MLPH was upregulated in PDAC to promote tumor metastasis, suggesting a novel function of MLPH in facilitating metastasis. Importantly, the mechanism by which Gln deficiency induces MLPH upregulation has not yet been elucidated. A recent study showed that *MLPH* expression is regulated by the glucocorticoid receptor (GR) in melanocytes [33]. GR, also known as NR3C1, is a transcription factor that belongs to the nuclear receptor superfamily. Under basal conditions, GR is localized in the cytoplasm; however, it translocates to the nucleus and regulates the expression of downstream target genes when bound to its ligands, cortisol and corticosterone, in humans and rodents, respectively [34]. Using in silico prediction, several glucocorticoid-responsive elements were identified in the promoter region of *MLPH* (data not shown), suggesting that GR regulates *MLPH* expression. Glucocorticoids facilitate the progression of pancreatic cancer [35] and promote breast cancer metastasis [36]. Active GR causes immunotherapy resistance in PDAC [37]. GR expression correlates with high PD-L1 expression and poor survival in pancreatic cancer. Thus, we hypothesize that in Gln deficient conditions, glucocorticoids activate GR to induce MLPH expression and PDAC metastasis. However, this hypothesis requires further confirmation by future studies.

Upregulation of MLPH promotes primary ciliogenesis; however, the underlying molecular mechanisms remain unclear. In our study, we found that MLPH was localized to the cytoplasm, and the centrosome. MLPH forms a heterotrimeric complex with myosin Va (MyoVa) and Rab27a to transport melanosomes via actin filaments or microtubule networks [22, 23]. MyoVa transports preciliary vesicles to the distal appendage to initiate primary ciliogenesis [38]. Our study showed that the depletion of MLPH inhibited primary ciliogenesis. Thus, we hypothesized that MyoVa interacts with MLPH and promotes its centrosomal targeting to induce primary ciliogenesis. Alternatively, MLPH may function as an adaptor of MyoVa to carry periciliary vesicles for the formation of primary cilia complexes. Based on the critical role of MLPH in facilitating PDAC metastasis, testing the potential therapeutic effect of the MLPH inhibitor 16-kauren (16-kauren-2b-18,19-triol) [39] may be worthwhile as a novel strategy for preventing PDAC metastasis.

Here, we showed that PLCG1 was activated and promoted PDAC EMT and invasion upon Gln deficiency. Although PLCG1 promotes metastasis in several cancers [40, 41], this is the first study showing that PLCG1 promoted PDAC metastasis. PLCG1 hydrolyzes phosphatidylinositol 4,5-bisphosphate (PIP_2_) into 1,2-diacylglycerol (DAG) and 1,4,5-inositol trisphosphate (IP_3_) followed by activation of the protein kinase C (PKC) cascade and promoting the release of intracellular Ca^2+^. Protein kinase C iota (PKCi) has been linked to pancreatic tumorigenesis [42]; however, its role in PDAC metastasis has not been examined clearly. In addition to PKC, calcium release-activated calcium (CRAC) channels, which are composed of stromal interaction molecule 1 (STIM1) and Orai1 proteins, are known to influence the growth and metastasis of PDAC [43]. We speculate that PLCG1 activation will increase either DAG or intracellular Ca^2+^ concentrations, followed by the activation of PKCi and CRAC channels, respectively, to promote PDAC metastasis. The underlying molecular mechanism needs to be elucidated so that an inhibitory molecule that blocks PLCG1 signaling can be tested as an adjuvant therapy to alleviate PDAC metastasis.

During pancreatic tumorigenesis, the number of primary cilia is gradually reduced. Therefore, the PDAC cells are devoid of primary cilia. Our recent study showed that PDAC cells regain the ability to grow primary cilia, leading to chemoresistance. In this study, we demonstrated that, upon Gln deficiency, PDAC cells regrow primary cilia, thus promoting PDAC metastasis in vitro and in vivo. Importantly, MLPH plays a critical role in mediating these events. Our studies imply that regrowth of primary cilia in PDAC is a poor prognostic biomarker linked to chemoresistance and metastasis. We believe that MLPH is a good candidate for the treatment of PDAC cells it is aberrantly upregulated and accumulates in the centrosomes to promote the regrowth of primary cilia. Importantly, this phenotype is restricted to metastatic PDAC. Therefore, we anticipate that this study will advance our understanding of the impact of tumor cell regrowth on primary cilia and provide novel information for designing a new therapeutic strategy.

## Materials and methods

### Cell cultures

The human pancreatic ductal adenocarcinoma (PANC-1) and murine pancreatic ductal adenocarcinoma (KPC) cell lines were maintained in Roswell Park Memorial Institute (RPMI)-1640 medium (Gibco, Grand Island, NY, USA) containing 10% fetal bovine serum (FBS) (Gibco) and 1% sodium pyruvate (Gibco). To establish long-term glutamine-deprived pancreatic cancer cells, PANC-1 and KPC cells were initially treated with glutamine-free RPMI-1640 medium (Gibco #21870076) containing 10% FBS and 1% sodium pyruvate. All cell lines were incubated at 37 °C in a humidified atmosphere containing 5% CO_2_. Cells were regularly examined for *Mycoplasma* contamination by immunofluorescence staining and immunoblotting. A randomization method was employed to assign subjects to different experimental groups. All samples and animals included in the study were retained for analysis. The phenotypic analysis was conducted in a blinded manner.

### Reagents

U-73122 (phospholipase C inhibitor; HY-13419) was purchased from MedChemExpress (Monmouth Junction, NJ, USA).

### RNA interference (RNAi)

IFT88 and CEP164 were depleted in human PANC-1 parental and -QQ cells using annealed siRNAs with the following target sequences:

siIFT88: 5′-cgacuaagugccagacucauu [dt] [dt]-3′ [44]

siCEP164: 5′-caggugacauuuacuauuuca [dt] [dt]-3′ [45]

Scrambled siRNA: 5′-gaucauacgugcgaucaga [dt] [dt]-3′ (Sigma-Aldrich, St. Louis, MO, USA).

PLCG1 was depleted in human PANC-1 and -QQ cells using SMARTpool siGENOME siRNAs with the following four target sequences:

siPLCG1: 5′-ccaaccagcuuaagaggaa-3′, 5′-gaagugaacauguggauca-3′, 5′-gagcagugccuuugaagaa-3′, and 5′-ccaagaaggacucggguca-3′

Scrambled siRNA: K-002800-C4-01 (Dharmacon, Lafayette, CO, USA)

For siRNA transfection, 6 μL of Lipofectamine 2000 (Invitrogen, Carlsbad, CA, USA) was mixed with 500 μL of Opti-MEM (Life Technologies, Grand Island, NY) for 5 min and then 100 nM siRNA in 500 μL of Opti-MEM was added to this mixture, which was then incubated at room temperature for 25 min before being layered onto cells in 1 mL of standard or glutamine-free RPMI-1640 medium. PANC-1 cells were harvested for further experiments at 72 h after transfection.

Lentiviruses were transfected into PANC-1 cells to deliver three different anti-MLPH shRNAs.

MLPH#1: TRCN0000319000, CCTTTAGGACAATGTTGTGTA

MLPH#2: TRCN0000319072, GTCTTCTGAGAGTCAGATCTT

Scrambled shRNA: TRCN0000208001, ACACTCGAGCACTTTTTGAAT

All lentiviruses were purchased from the RNAi Core Laboratory of the Genomics Research Center, Academia Sinica, Taipei, Taiwan.

### Transwell invasion and migration assay

In vitro migration and invasion assays were examined using a Transwell cell culture insert (8 μm, 1 × 10^5^ pores/cm^2^, Falcon #303597, NY, USA) with the 24-well plate. A standard medium containing 10% FBS was added to the lower chamber. For the invasion assay, 100 μL/well Matrigel (Corning, New York, NY, USA) was first gently placed on the upper filters. Then, cells (3 × 10^4^ cells/100 μL) were resuspended with serum-free medium and seeded into the upper chamber. All the cell lines were incubated at 37 °C in a humidified atmosphere containing 5% CO_2_ for 12 h. Next, for fixation, the chambers were placed in another 24-well plate containing 100% methanol for 5 min, and the Transwell inserts were buckled on the tissue to volatilize the residual methanol. Next, the Transwell inserts were transferred to another 24-well plate containing 20% Giemsa (Merck #109204, Darmstadt, Germany) for two hours to stain the cells. After staining, the Transwell inserts were washed twice with water, and a cotton swab was used to remove the residual Matrigel and cells inside the Transwell inserts. Finally, invading and migrating cells were counted in random microscopic fields.

### EdU assay

Cells were seeded onto coverslips. After treatment and incubation, the medium was replaced with EdU labeling solution (10 µM in RPMI-1640 medium) and the cells were incubated at 37 °C in a humidified atmosphere of 5% CO_2_ for one hour. After labeling cells with EdU, cells were fixed with ice-cold methanol at −20 °C for 10 min, then wash with 1X phosphate buffered saline (PBS) three times and rocked for 15 min. Next, 80 μL of Click-iT® reaction cocktail (Invitrogen #C10269) was added to each coverslip followed by incubation at room temperature protected from light for 1 h, while the nuclei were stained with 6-diamino-2-phenylindole (DAPI; 0.1 μg/mL). After washing three times with PBS, the coverslips were mounted on glass slides in 50% glycerol. The prepared cells were observed using an Axio Imager M2 fluorescence microscope (Zeiss, Switzerland) and images were captured using ZEN pro software (Zeiss, Switzerland).

### Immunofluorescence microscopy

Cells were seeded onto coverslips. After treatment and incubation, cells were fixed with ice-cold methanol at −20 °C for 10 min, then washed with PBS three times and rocked for 30 min. Next, the cells were blocked with 5% BSA for 1 h at room temperature, followed by incubation with primary antibodies at 4 °C for 8 h. Next, the cells were washed three times with PBS. The cells were then incubated with fluorescein isothiocyanate-conjugated or Cy3-conjugated secondary antibodies at room temperature for 1 h in the dark, while the nuclei were stained with 0.1 μg/mL DAPI. After washing three times with PBS, the coverslips were mounted on glass slides in 50% glycerol. The prepared cells were observed and images captured as previously described.

### Immunoblotting assay

Cells were lysed using the CelLytic M cell lysis reagent (Sigma-Aldrich) with protease cocktail inhibitors (Roche, Mannhein, Germany) on ice for 10 min. Next, the lysates were centrifuged at 13,300 rpm for 10 min at 4 °C. Cell lysates were collected and proteins were quantified using the Bradford protein assay (Bio-Rad, Hercules, CA, USA). The quantified lysates were mixed with sample buffer and heated at 100 °C for 15 min. Next, the prepared samples were loaded onto gels and separated by SDS-polyacrylamide gel electrophoresis. After gel separation, the samples were transferred to polyvinylidene difluoride (PVDF) membranes at 20 V for 720 min in a cold room. After washing with TBST, membranes were blocked with 3% BSA in TBST at room temperature for 1 h. Next, the membranes were incubated with primary antibodies at 4 °C for 12 h. After extensive washing with TBST (three times), the membranes were incubated with horseradish peroxidase (HRP)-conjugated secondary antibodies at room temperature for 1 h. After washing with TBST three times, the signals were detected by ECL Detection Reagents. The following primary antibodies were used: anti-p-ERK (#4370), anti-ERK (#4695), anti-MMP2 (#40994), anti-E-cadherin (#3195), anti-N-cadherin (#13116), anti-Ku70 (#4588), anti-Rab27a (#69295), anti-CX43 (#3512), anti-GSK3α/β (#5676), anti-p-JNK (#4668), anti-JNK (#9252), anti-β-catenin (#9562), and anti-PLCγ-1 (#2822) antibodies were purchased from Cell Signaling Technology (Beverly, MA, USA). Anti-IFT88 (13967-1-AP), anti-ARL13B (17711-1-AP), anti-MLPH (10338-1-AP), and anti-MMP9 (10375-2-AP) antibodies were purchased from Proteintech (Chicago, IL, USA). anti-CEP164 (NBP1-81445) and anti-myosin 5a (NBP1-92156) antibodies were purchased from Novus (Littleton, CO, USA). anti-β-actin (AC-15; GTX26276) and anti-PLCγ-1 (phospho-Y783; GTX24828) were purchased from GeneTex (Irvine, CA, USA). Anti-acetylated tubulin (T6793) was purchased from Sigma-Aldrich. Anti-CP110 (ab99338) antibody was purchased from Abcam (Cambridge, UK).

### Quantitative reverse transcription PCR

Total RNA was extracted from the cell samples using TRIzol reagent (Invitrogen). The cDNA synthesis was carried out using SensiFAST^™^ Real-Time PCR Kits (Applied Biosystems, Waltham, MA). Quantitative real-time PCR (qRT-PCR) was performed using SYBR Green Real-time PCR Master Mix (Applied Biosystems) and the StepOnePlus Real-Time PCR system (Applied Biosystems). Relative abundance of mRNA expression was normalized to human glyceraldehyde 3-phosphate dehydrogenase (GAPDH) gene expression as the internal reference and based on the 2^-ΔΔCt^ equation. The qPCR primer sequences used were listed in the Supplementary Table. S1.

### Spheroid generation

Multicellular tumor spheroids derived from PANC-1 cells were generated in culture dishes containing microwells provided by Dr. Tu [46]. Before use, microwells were sterilized by incubation in 70% ethanol, exposed to UV light for 12 h, and rinsed twice with PBS. Then, the microwells were immersed in 0.2% Pluronic F127 (Sigma-Aldrich) in 1x PBS for 40 min. Next, untreated or treated cells (3.5×10^5^ cells) were seeded into microwells and incubated at 37 °C in a humidified atmosphere of 5% CO_2_. After 30 min, fresh medium was replaced in the microwells. The cells proliferated and aggregated into mature MCTSs after five days of incubation.

### Spheroid invasion assay

Mature MCTSs were first transferred from the microwell dish by gentle pipetting on the dish surface. Next, these MCTSs were filtered through 70 μm cell strainers (GeneDireX, US) to avoid non-mature MCTSs and single cells. The MCTSs were then centrifuged at 500 rpm for 3 min at room temperature. Fresh standard or glutamine-free RPMI-1640 medium was added to resuspend the MCTSs, followed by removal of the culture medium. Next, MCTSs were embedded in the collagen matrix (10 MCTSs/10 µl/well). The collagen matrix contained 2 mg/mL collagen solution (Corning® Collagen I, Rat Tail), PBS, and was adjusted to pH 7.0 with 1 N NaOH. MCTSs and collagen mixture were seeded on the dish and further incubated at 37 °C in a humidified atmosphere of 5% CO_2_ for 30 min. Fresh medium was added until a solid gel formed that covered the domes.

### Kinase array

The Human Phospho-Kinase Array (R&D Systems, Minneapolis, US, Catalog # ARY003B) was used to analyze the phosphorylation profiles of kinases and their protein substrates. Cells were lysed using CelLytic M cell lysis reagent (Sigma-Aldrich) with a protease inhibitor cocktail (Roche) on ice for 30 min. Next, the lysates were centrifuged at 13,300 rpm for 5 min at 4 °C. Cell lysates were collected and proteins were quantified using the Bradford protein assay (Bio-Rad). The quantified lysates were adjusted to 500 μg. The membranes were blocked with array buffer 1 for 1 h, removed, replaced with the prepared protein samples, and incubated overnight in a cold room. Next, each membrane was washed thrice with washing buffer for 10 min. Each membrane was incubated with a detection antibody cocktail for 2 h at room temperature on a rocking platform. After incubation, the membranes were washed three times with washing buffer for 10 min each. Membranes were then incubated with streptavidin-HPR for 30 min at room temperature on a rocking platform shaker. After washing three times with washing buffer, the signals were detected using the Chemi Reagent Mix.

### RNA-Seq analysis

Total RNA was extracted from the cell samples using TRIzol reagent (Invitrogen). cDNA libraries were prepared from isolated total RNA using the TruSeq Stranded mRNA Library Prep Kit (Illumina, San Diego, CA, USA), and library quality was assessed using an Agilent BioAnalyzer 2100 (Agilent Technologies, Santa Clara, CA, USA). Sequencing was performed on an Illumina NovaSeq 6000 using paired-end sequencing at Genomics, Bioinformatics, and Single Cell Core, Faculty of Health Sciences, University of Macau.

### Gelatin zymography

Cells were lysed using the CelLytic M cell lysis reagent (Sigma-Aldrich) with protease cocktail inhibitors (Roche) on ice for 10 min. Next, the lysates were centrifuged at 13,300 rpm for 10 min at 4 °C. Cell lysates were collected and proteins were quantified using the Bradford protein assay (Bio-Rad). The quantified lysates were mixed with sample buffer, loaded onto gels, and separated using 1% gelatin SDS–PAGE. After washing three times with water, the gel was transferred to a developing buffer and incubated at room temperature for 30 min. Next, fresh developing buffer was added and the gel was incubated at 37 °C overnight. After extensive washing with water thrice, the gel was incubated with 0.5% Coomassie blue staining buffer for 1 h. After washing with the de-staining buffer, data were captured using a camera.

### Orthotopic animal models

Five-week-old male NOD-SCID or C57BL/6 J mice were used for pancreas orthotopic injection experiments. Before injection, luciferase-expressing PANC-1 or KPC parental, -QQ (shScr), and MLPH-depleted -QQ (shMLPH) cells were generated under non-treated Gln-starvation conditions for three months in the presence or absence of MLPH knockdown. PANC-1 or KPC cells, 3 × 10^6^, suspended in 100 μL of RPMI medium were injected into the pancreas head of mice (*n* = 5 for each group). After seven weeks, the tumor growth and metastatic abilities were monitored using an in vivo imaging (Xenogen IVISR Spectrum Noninvasive Quantitative Molecular Imaging System, PerkinElmer, Waltham, Massachusetts, US), followed by intraperitoneal injection of 100 μL D-luciferin (15 mg/mL in PBS, Promega, Madison, WI, USA, #P1043). After measuring whole-body luciferase signals, mice were sacrificed, primary injected tumor xenografts and metastatic organs were resected, and luciferase signals were measured. All animals were randomly assigned to the groups. No blinding was performed during the xenograft. All animals included in the study were retained for analysis. All animal experiments were performed in compliance with the National Cheng Kung University Laboratory Animal Center’s Institutional Guidelines for the Use and Care of Animals and all animal experiment protocols were reviewed and approved by the relevant animal ethics committee.

### Study population and clinical informatics collection

A total of 97 postoperative pancreatic cancer specimens were retrieved for bulk RNA sequencing from the NCKUH in Taiwan. Details regarding the clinical characteristics of these patients are provided in supplementary Table. S2. This study was approved by the Institutional Review Board (IRB) of the NCKUH (IRB number: NCKUH B-ER-110-420) and was conducted in accordance with the ethical research guidelines. Patient demographics and clinicopathological parameters between November 2013 and March 2023 were reviewed using electronic medical records. Outcome measures included OS and DFS. OS was calculated from the date of resection to the date of death or last follow-up. The DFS was calculated from the date of resection to the date of disease progression, last follow-up, or death.

### Tissue preparation and bulk RNA sequencing

Fresh-frozen cancer tissues were homogenized using the QIAzol Lysis Reagent (Qiagen, Venlo, Netherlands). Total RNA was isolated using the miRNeasy Mini Kit (Cat No. 217004, Qiagen). Transcriptome sequencing experiments included RNA extraction, quality control (QC), library construction, purification, library QC, quantitation, sequencing cluster generation, and high-throughput sequencing. To ensure the accuracy and reliability of the analysis results, every step was strictly monitored, and QC was performed. After mixing the libraries based on their effective concentrations and required sequencing data volume, the Illumina platform was used for high-throughput sequencing. The filtered data were subsequently aligned to the reference genome used in the short-read alignment using Hisat2 (v2.0.1) [47] with default parameters. The level of gene expression was measured by read density; the higher the read density, the higher the gene expression level. Gene expression was calculated using the following formula: fragments per kilobase per million reads (FPKM), based on read counts from HT-seq (V 0.6.1) [48].

$$\frac{{\rm{Total}}\; {\rm{exon}}\; {\rm{fragments}}}{{\rm{FPKM}}=\,{\rm{Mapped}}\; {\rm{reads}}({\rm{millions}}){\rm{x}}\; {\rm{exon}}\; {\rm{length}}\,({\rm{KB}})}$$

### Statistical analysis

All statistical data were generated using the SPSS software (version 17.0; SPSS, Chicago, IL, USA) and Prism 5.0 software (GraphPad Software, La Jolla, CA). All results are presented as mean ± standard deviation from at least three independent experiments, and more than 500 cells were counted in each individual group. Error bars in the bar plots represent the standard error of the mean of at least three independent experiments. Differences between two groups were compared using unpaired two-tailed t-tests and ANOVA for multi-group comparisons, for which a *P* value < 0.05 was considered statistically significant. The Chi-square test was used to detect differences in patient characteristics between patients with low and high MLPH levels. The optimal cutoff value for MLPH was calculated using Youden’s index. The survival rates of patients were calculated using the Kaplan–Meier method, and the differences between curves were evaluated using the log-rank test. The results are presented as hazard ratios with 95% confidence intervals (95% CI). Pearson’s correlation analysis was performed to analyze the correlation between the MLPH and IFT43. Statistical significance was set at *P* < 0.05.

## Supplementary information

SUPPLEMENTAL MATERIAL

Original data

## Data Availability

All data supporting the findings of this study are presented in this published article and its Supplementary information files.
