# Supplementary material for: Melanophilin-induced primary cilia promote pancreatic cancer metastasis
Source: Cell Death Dis. 2025 Jan 16;16(1):22. doi: 10.1038/s41419-025-07344-2 (PMC11739566; doi:10.1038/s41419-025-07344-2)
Supplement: Supplementary file 1 — SUPPLEMENTAL MATERIAL [file 41419_2025_7344_MOESM1_ESM.docx]

**Supplemental information**


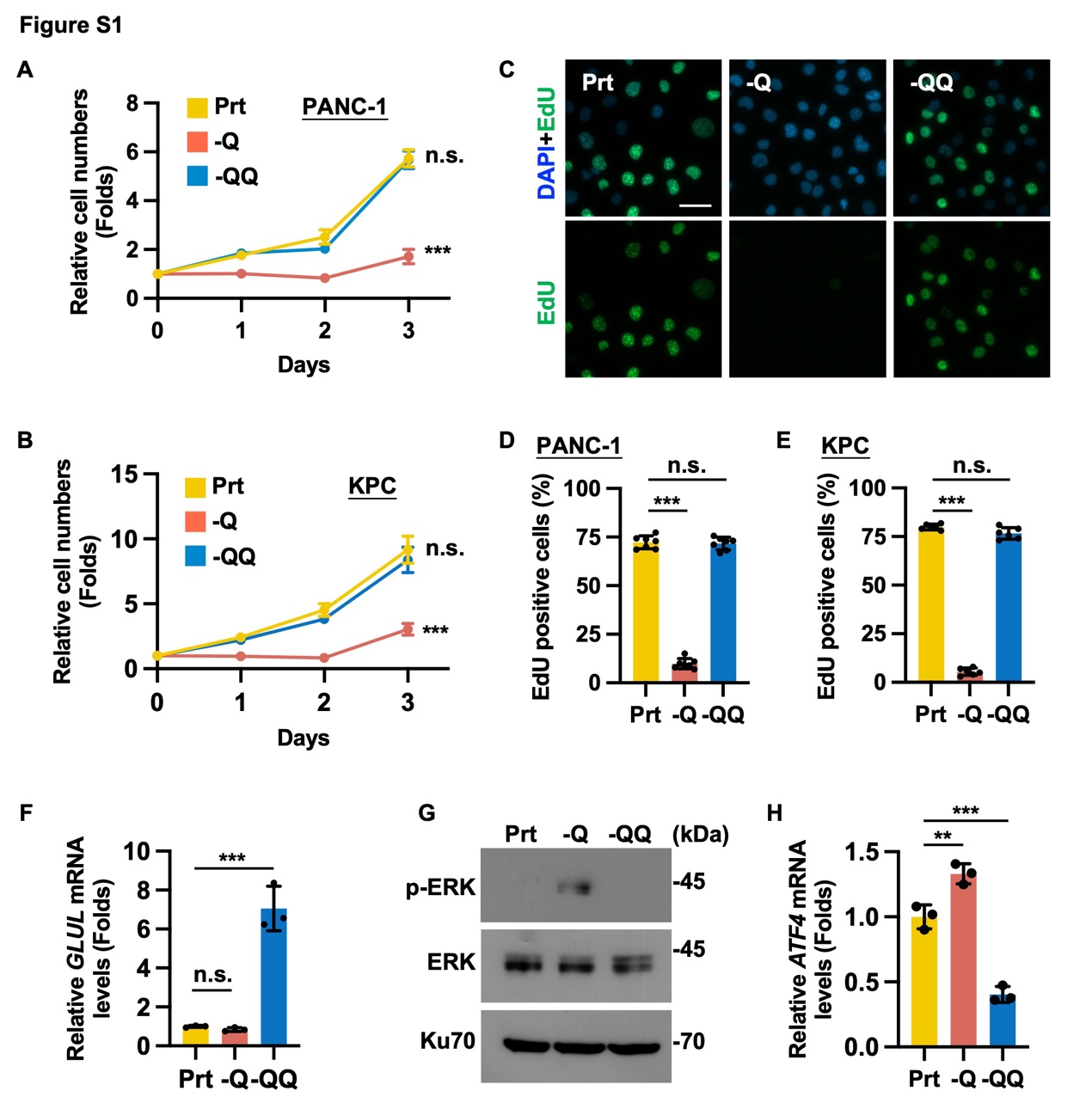


**Figure S1. PDAC cells regain the ability to proliferate under long-term Gln deficiency.**

(A-B) Short-term Gln deprivation (3 days, -Q) inhibited cell growth; however, long-term Gln deprivation (3 months, -QQ) regained the ability to proliferate in (A) PANC-1 and (B) KPC cells. Quantitative results of relative cell numbers after seeding for 1, 2, or 3 days. Prt: parental cells. (C-E) -QQ cells regained the ability to enter S phase. (C) EdU-positive cells (green) were detected by immunofluorescence staining. DNA was stained with DAPI (blue). Scale bar, 50μm. (D-E) Quantitative results of the proportion of EdU-positive cells in (D) PANC-1 or (E) KPC cells. (F) GLUL expression was upregulated in -QQ cells. Quantitative results of relative mRNA levels of *GLUL* in Prt, -Q, or -QQ PANC-1 cell. (G) ERK was activated in -Q cells. Extracts of Prt, -Q, and -QQ PANC-1 cells were analyzed by western blot assay with antibodies against phosphorylated ERK, ERK, and Ku70 (loading control). (H) ATF4 was downregulated in -QQ cells. Quantitative results of relative mRNA levels of *ATF4* were analyzed in Prt, -Q, or -QQ PANC-1 cells. Data are represented as the mean ± SD of three independent experiments. n.s. no significance, ** P<0.01, *** P<0.001.


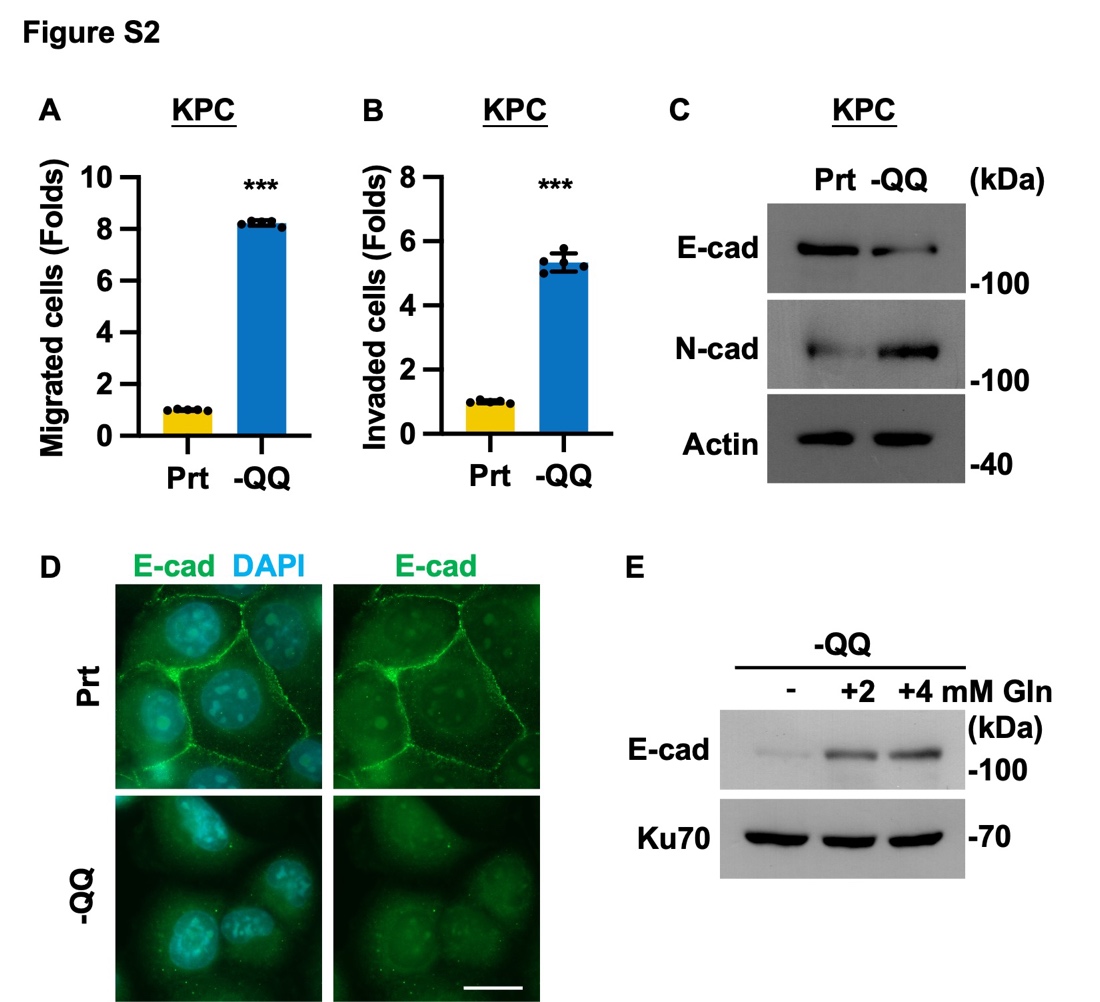


**Figure S2. Gln deficiency induces PDAC EMT.**

(A-B) Gln-deficient condition promoted KPC cells migration and invasion. The cell invasion and migration ability of parental (Prt) cells and -QQ KPC cells were evaluated using Trans-well analysis. (A) Quantitative results of the relative migrated cell numbers. (B) Quantitative results of the relative invaded cell numbers. (C) Gln deficiency induced EMT of KPC cells. Extracts of parental (Prt) and -QQ KPC cells were analyzed by western blot assay with antibodies against E-cadherin (E-cad), N-cadherin (N-cad), and actin. (D) Gln deficiency induced EMT of PANC-1 cells. Immunofluorescence staining of Prt or -QQ PANC-1 cells with antibodies against E-cad (green). DNA was stained with DAPI (blue). Scale bar, 40μm. (E) Supplementation of Gln restored the E-cadherin expression in -QQ cells. Extracts of -QQ PANC-1 cells supplemented with 0, 2, 4 mM Gln were analyzed by western blot assay with antibodies against E-cad and Ku70. Data are represented as the mean ± SD of three independent experiments. *** P<0.001.


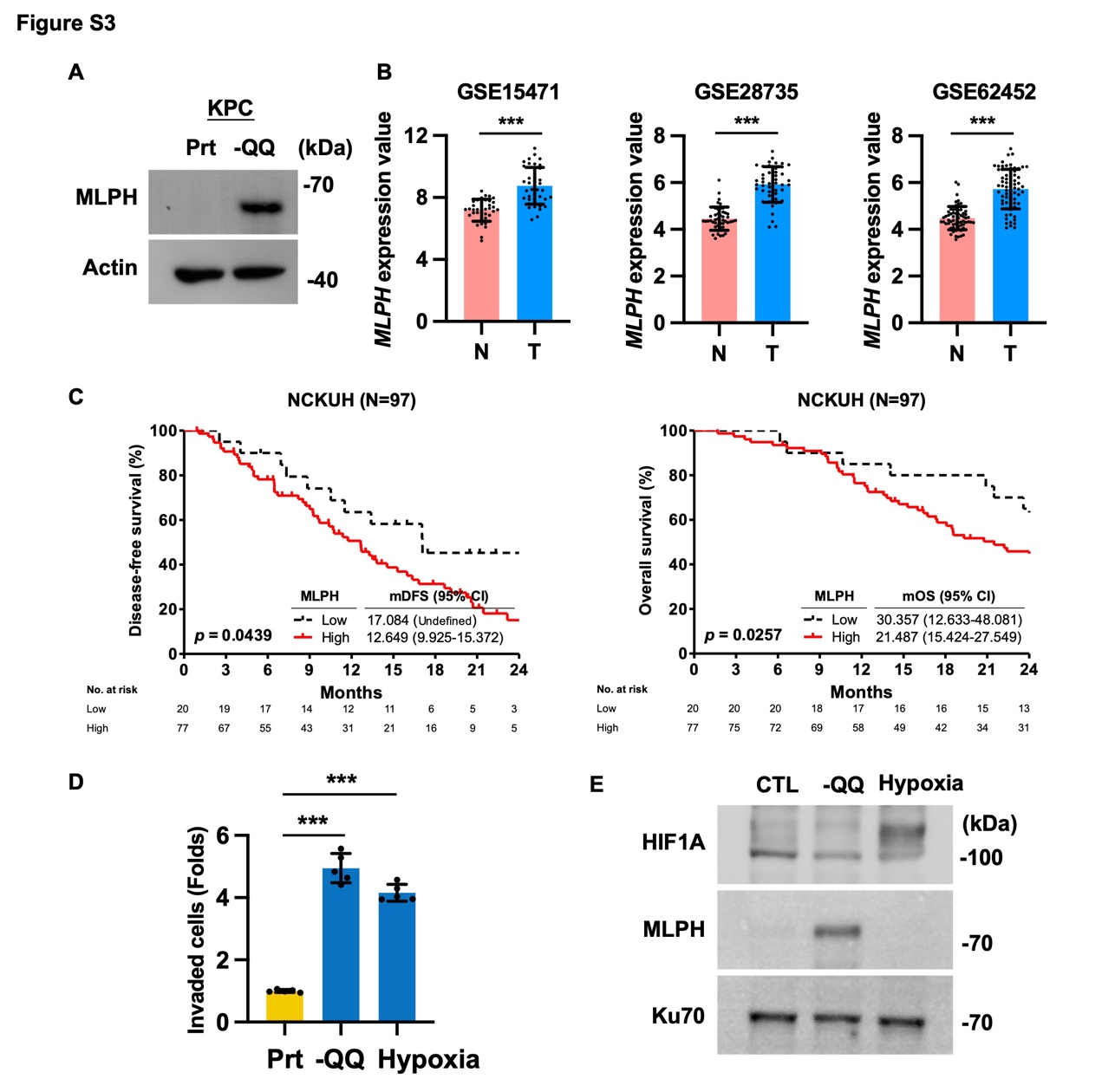


**Figure S3. High MLPH expression correlates with poor prognosis in PDAC patients.**

(A) MLPH was upregulated in -QQ KPC cells. Extracts of Prt and -QQ KPC cells were analyzed by western blot assay with antibodies against MLPH and Ku70 (loading control). (B) MLPH is upregulated in PDAC tumor tissues compared to benign pancreas tissue in PDAC patients. The mRNA gene expression of MLPH in normal (N) and tumor (T) were obtained from GEO database: GSE15471, GSE28735, and GSE62452. (C) Disease-free survival and overall survival analysis using the Kaplan–Meier method indicated that patients with high MLPH protein expression had poorer survival than others. Data were analyzed from the NCKUH cohort. (D) Gln deprivation and hypoxia promoted PDAC invasion. Quantitative results of the relative invaded cell numbers in parental (Prt), hypoxia-treated cells, and -QQ PANC-1 cells. (E) MLPH was upregulated in Gln deprivation but not under hypoxia conditions. Extracts of Prt, -QQ, and hypoxia-treated PANC-1 cells were analyzed by western blot assay with antibodies against HIF1A, MLPH, and Ku70 (loading control). P values were determined using the log-rank test. *** P<0.001.


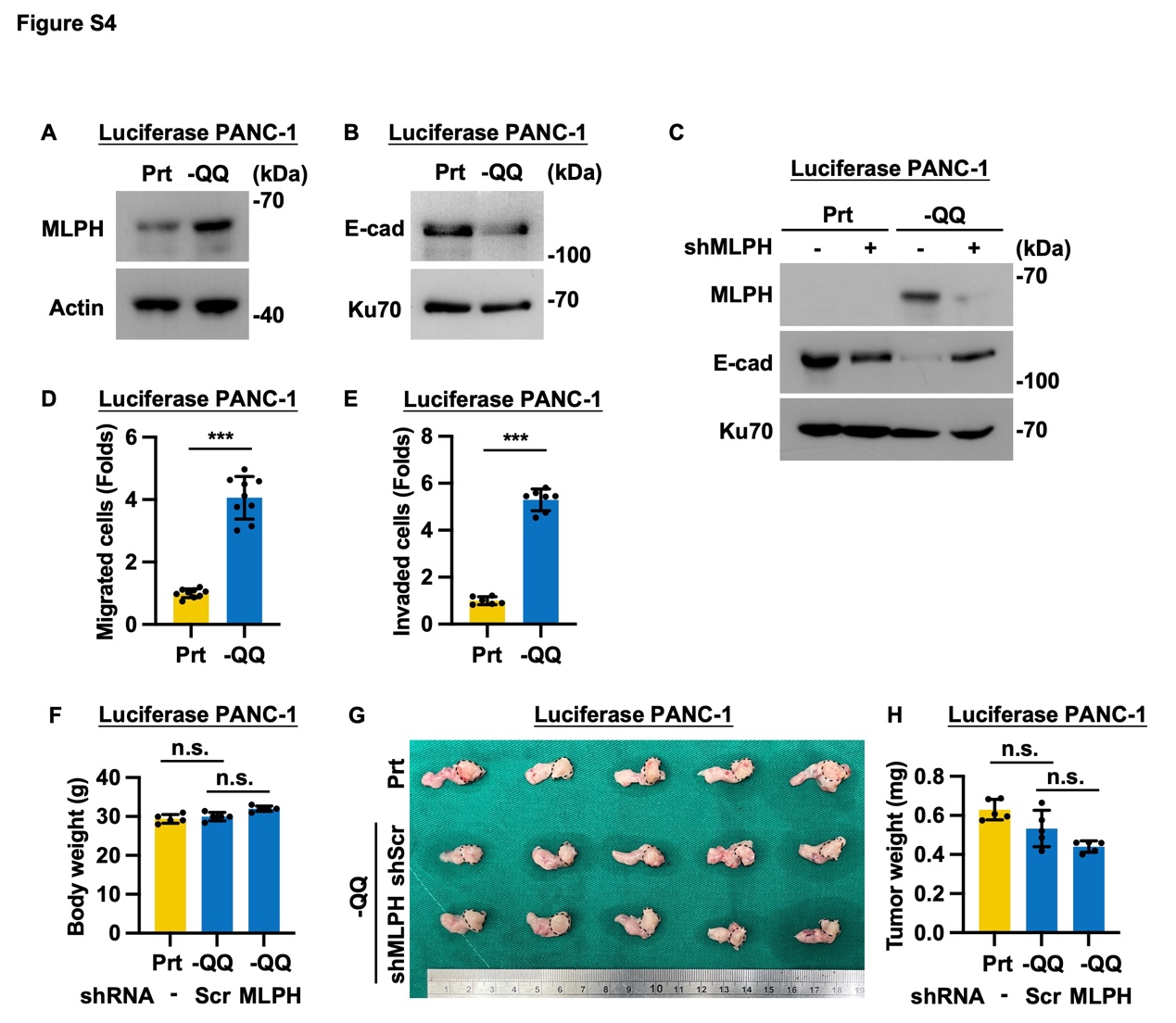


**Figure S4. Gln deprivation promotes EMT, migration, and invasion in luciferase-expressing PANC-1 cells.**

(A-C) MLPH regulated EMT in luciferase-expressed -QQ PANC-1 cells. (A-B) Upregulation of MLPH and downregulation of E-cadherin were observed in luciferase-expressed -QQ PANC-1 cells. Extracts of parental (Prt) or -QQ cells were analyzed by western blot assay with antibodies against MLPH, E-cadherin (E-cad), Ku70, and actin. (C) Depletion of MLPH decreased EMT in luciferase-expressed -QQ PANC-1 cells. Extracts of Prt or -QQ luciferase-expressed -QQ PANC-1 cells in the absence or presence of shRNA against MLPH (shMLPH#1) were analyzed by western blot with antibodies against MLPH, E-cad, and Ku70. (D-E) Gln deficiency promoted cell migration and invasion in luciferase-expressed -QQ PANC-1 cells. Quantitative results of the relative migrated (D) and invaded (E) cell numbers in Prt and -QQ luciferase-expressing PANC-1 cells. (F-H) MLPH did not affect tumor sizes in the orthotopic mouse model. Orthotopic injection of luciferase-expressed PANC-1 parental (Prt), -QQ (shScr), or MLPH depleted-QQ cells (shMLPH#1) did not affect (F) mouse body weight and (G-H) pancreatic tumor sizes. n=5 in each group. Data are represented as the mean ± SD of three independent experiments. n.s. no significance, *** P<0.001.


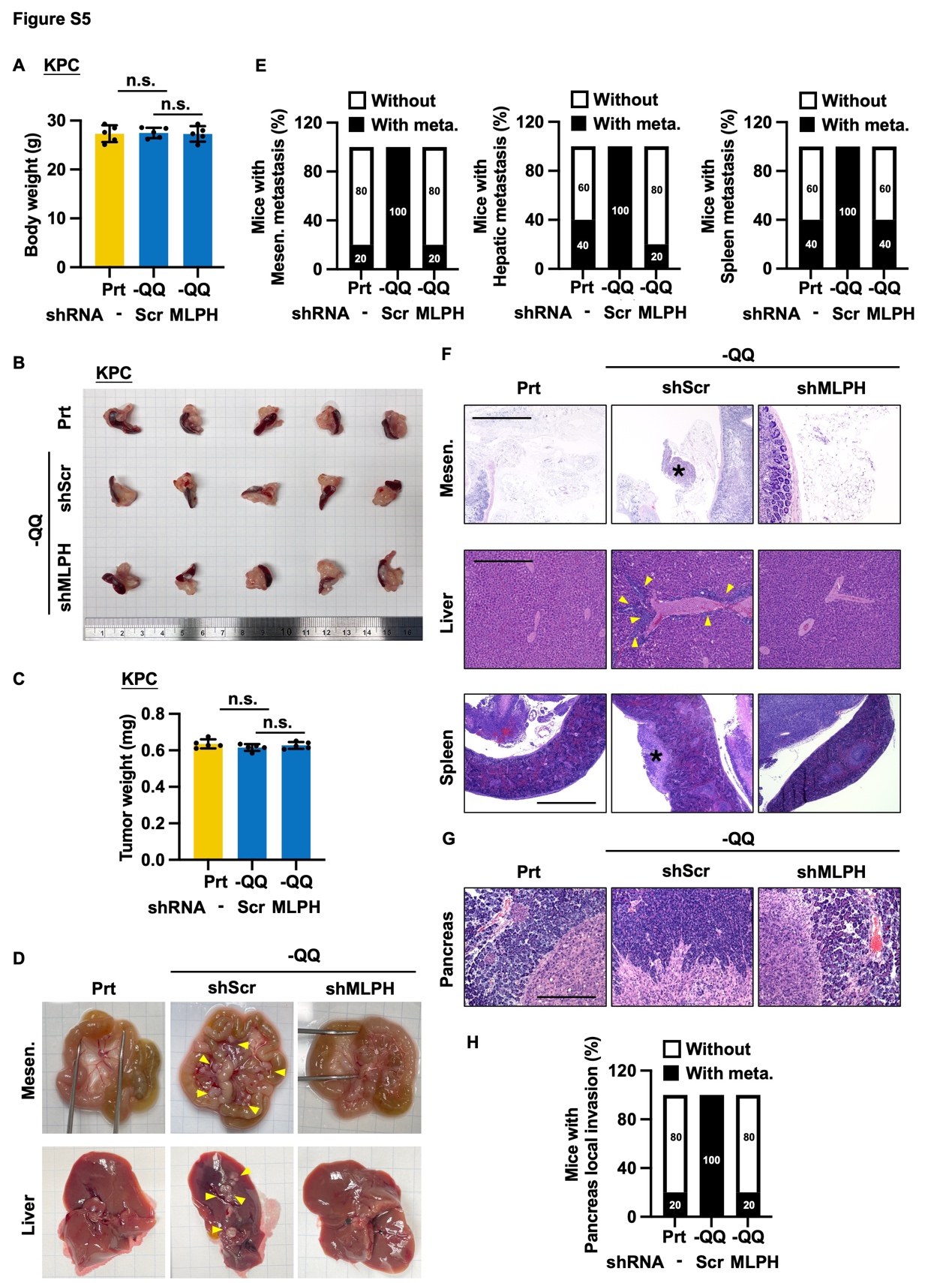


**Figure S5. MLPH upregulation promotes PDAC metastasis under Gln deficiency in the orthotopic** **immunocompetent C57BL/6 J mouse model.**

(A-C) MLPH did not affect tumor sizes in the orthotopic mouse model. Orthotopic injection of murine KPC parental (Prt), -QQ (shScr), or MLPH depleted-QQ cells (shMLPH#1) did not affect (A) mouse body weight and (B-C) pancreatic tumor sizes. (D) Metastatic nodules localized on the mesenteries (Mesen.), liver, and spleen in -QQ (shScr) groups but disappeared in MLPH depleted-QQ (shMLPH) groups. Metastatic colonies were labeled as arrowheads. (E) Quantitative relative percentages of metastasis of mesenteries (Mesen.), liver, and spleen of Prt, -QQ-shScr, and -QQ-shMLPH groups. (F) Representative H&E staining of the mesenteries (Mesen.), liver, and spleen of Prt, -QQ-shScr, and -QQ-shMLPH groups. Metastatic colonies were labeled as arrowheads and asterisks. Scale bar, 100 μm. (G-H) MLPH promoted pancreatic local invasion. (G) Representative H&E staining of the pancreas of Prt, -QQ-shScr, and -QQ-shMLPH groups. Scale bar, 100 μm. (H) Quantitative results of pancreatic local invasion were calculated in Prt, -QQ-shScr, and -QQ-shMLPH groups. n=5 in each group. Data are represented as the mean ± SD of three independent experiments. n.s. no significance.


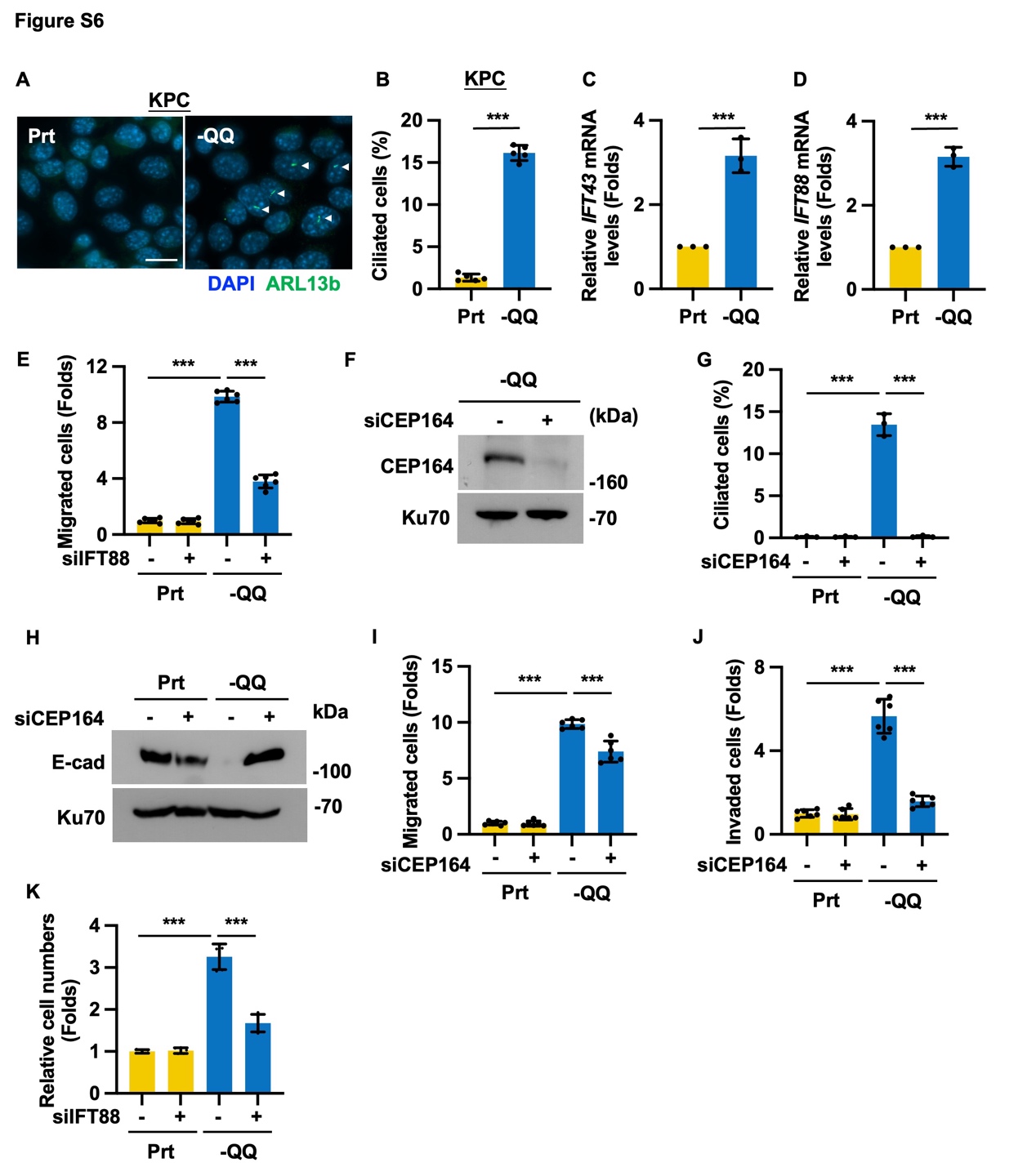


**Figure S6. Primary cilia participate in PDAC EMT and invasion under Gln deficiency.**

(A-B) Primary ciliogenesis occurred in -QQ KPC cells. (A) Primary cilia were detected by immunofluorescence staining with an antibody against ARL13b (green) in parental (Prt) or -QQ KPC cells. DNA was stained with DAPI (blue). Scale bar, 10 µm. (B) Quantitative results of the frequency of ciliated cells in (A). (C-D) Upregulation of ciliary genes in -QQ cells. Quantitative results of relative mRNA levels of IFT43 (C) and IFT88 (D) in the Prt or -QQ PANC-1 cells. (E-J) Primary cilia promoted EMT, cell migration, and invasion. (E) Quantitative results of the relative migrated cells in Prt or -QQ PANC-1 cells in the presence or absence of siRNA against IFT88 (siIFT88). (F) CEP164 was efficiently depleted. Extracts of -QQ PANC-1 cells in the absence or presence of siRNA against CEP164 (siCEP164) were analyzed by western blot assay with antibodies against CEP164 and Ku70. (G) Depletion of CEP164 inhibited primary ciliogenesis. Quantitative results of the frequency of ciliated cells in Prt or -QQ PANC-1 cells in the absence or presence siCEP164. (H) Depletion of CEP164 restored E-cadherin expression. Extracts of Prt or -QQ PANC-1 cells in the absence or presence siCEP164 were analyzed by western blot assay with antibodies against E-cad and Ku70. (I-J) Depletion of CEP164 inhibited cell migration and invasion. Quantitative results of relative (I) migrated or (J) invaded cells were measured in Prt or -QQ PANC-1 cells in the absence or presence siCEP164. (K) Primary cilia protect PDAC cells against gemcitabine treatment under Gln deprivation. Quantitative results of relative cell numbers in gemcitabine-treated Prt or -QQ PANC-1 cells in the presence or absence of siRNA against IFT88 (siIFT88). Data are represented as the mean ± SD of three independent experiments. *** P<0.001.


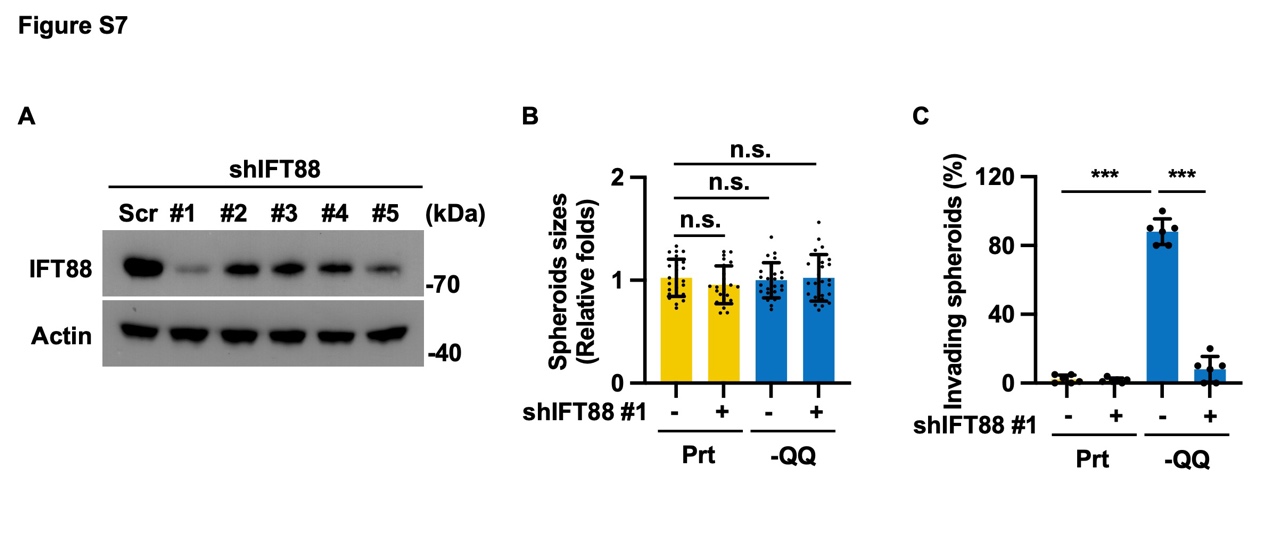


**Figure S7. Primary cilia induce invaded spheroids.**

(A) IFT88 was efficiently depleted. Extracts of PANC-1 cells infected with different sequences of shRNA against IFT88 (#1-#5) or scramble control (shScr) were analyzed by western blot with antibodies against IFT88 and actin. (B) Depletion of IFT88 did not affect the average size of spheroids. Quantitative results of the relative size of the spheroids in Prt and -QQ in the absence or presence of shRNA against IFT88 (shIFT88#1) were measured. (C) Depletion of IFT88 reduced the proportion of invading spheroids. Quantitative results of the invading spheroids in Prt or -QQ in the absence or presence of shIFT88#1. Data are represented as the mean ± SD of three independent experiments. n.s. no significance, *** P<0.001.


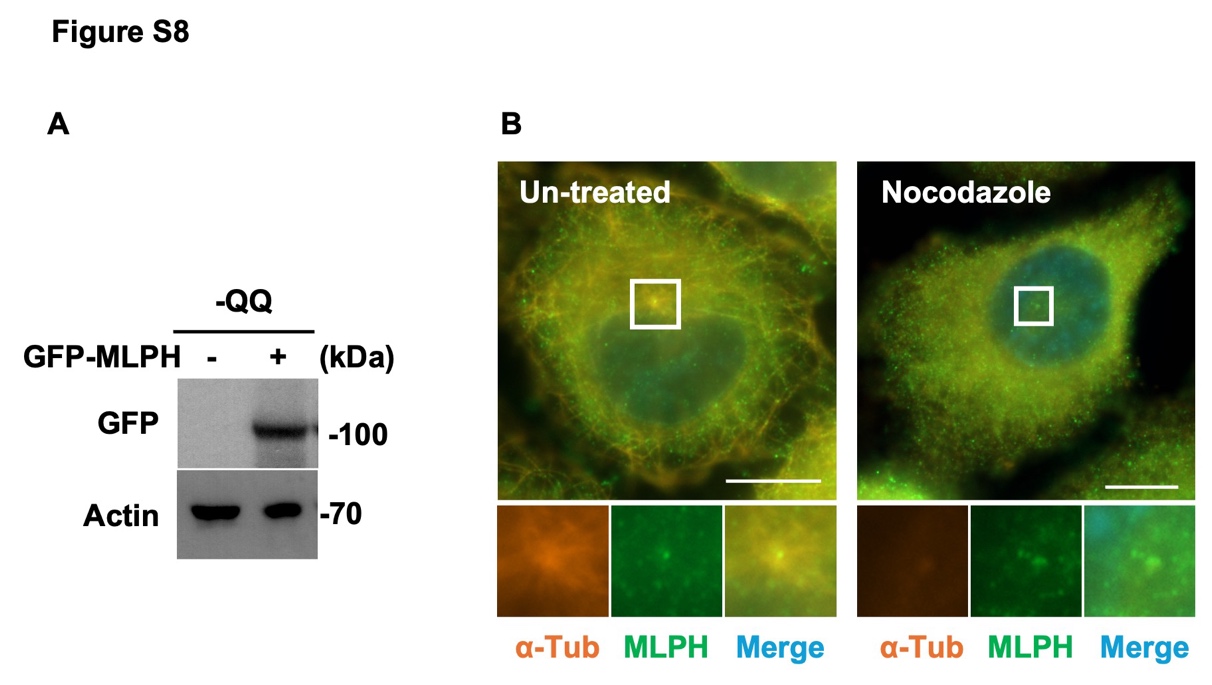


**Figure S8. The recruitment of MLPH is independent of microtubule assembly.**

(A) Overexpression of GFP-tagged MLPH in -QQ cells. Extracts of -QQ PANC-1 cells transfected with GFP or GFP-tagged MLPH were analyzed by western blot assay with antibodies against GFP and actin. (B) Microtubule inhibitor (Nocodazole) did not affect MLPH recruitment. Immunofluorescence staining of -QQ PANC-1 cells treated with or without Nocodazole with antibodies against MLPH and α-Tub. DNA was stained with DAPI (blue). Scale bar, 10μm.


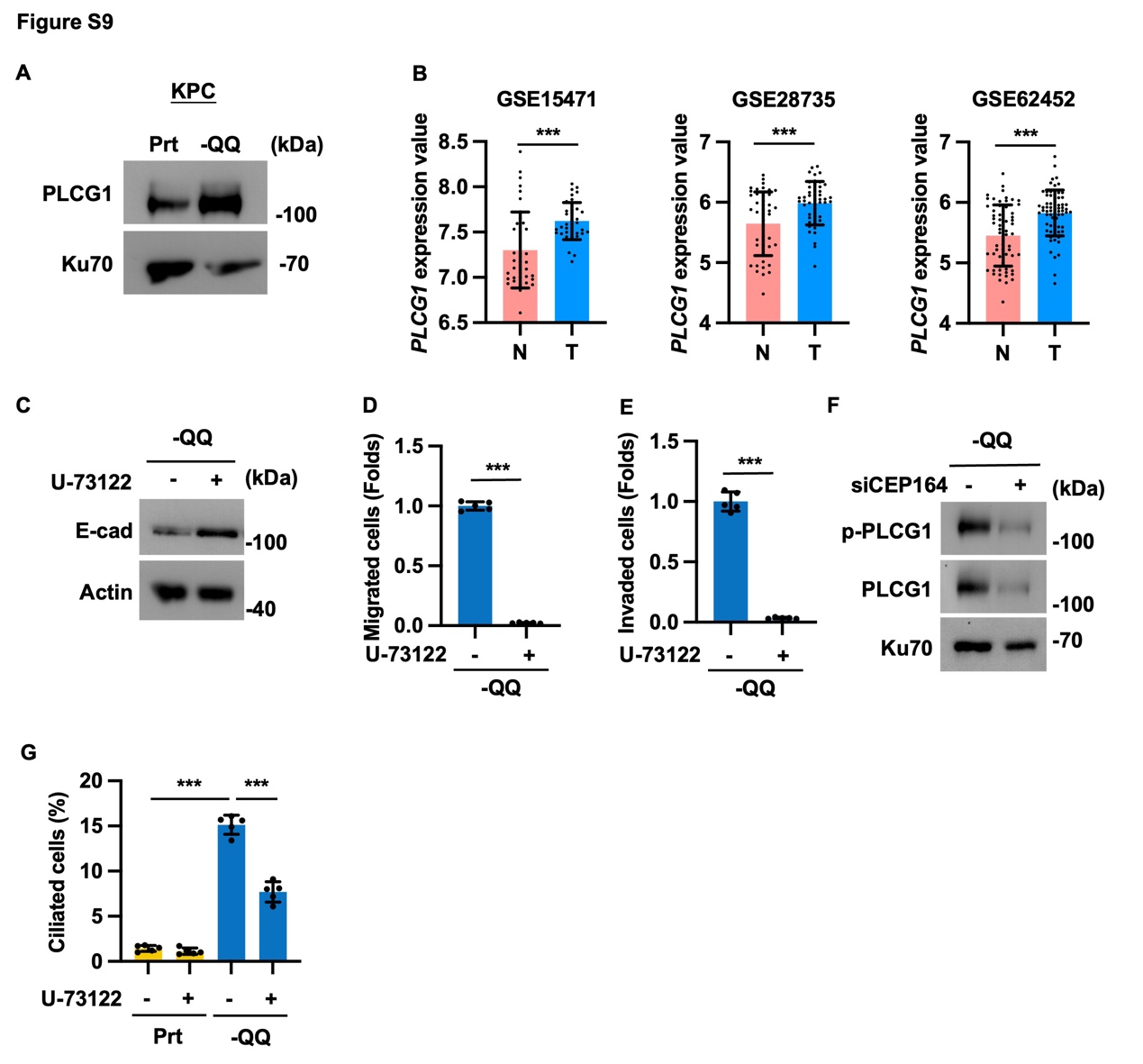


**Figure S9. PLCG1 promotes EMT, migration, and invasion upon Gln deficiency.**

(A) PLCG1 was upregulated in -QQ KPC cells. Extracts of parental (Prt) or -QQ KPC cells were analyzed by western blot assay with antibodies against PLCG1 and Ku70. (B) PLCG1 mRNA expression was upregulated in tumor tissues of patients with PDAC. The mRNA gene expression of PLCG1 in normal (N, left) and tumor (T, right) tissues were analyzed. Microarray datasets were obtained from the Gene Expression Omnibus (GEO) database: GSE15471, GSE28735, and GSE62452. (C-E) PLCG1 inhibitor (U-73122) reduced PDAC EMT, cell migration, and invasion in -QQ cells. (C) Extracts of -QQ PANC-1 cells in the absence or presence of PLCG1 inhibitor U-73122 were analyzed with antibodies E-cad and actin. (D-E) Quantitative results of the relative migrated (D) and invaded (E) cell numbers in -QQ PANC-1 cells in the absence or presence of U-73122. (F) Depletion of CEP164 decreased PLCG1 expression. Extracts of -QQ PANC-1 cells in the absence or presence of siRNA against CEP164 (siCEP164) were analyzed by western blot assay with antibodies against phosphorylated PLCG1 at Y783 residues, PLCG1, and Ku70. (G) Inactivation of PLCG1 inhibited primary ciliogenesis. Quantitative results of the proportion of ciliated Prt or -QQ PANC-1 cells in the absence or presence of U-73122 were measured. Data are represented as the mean ± SD of three independent experiments. *** P<0.001.

**Table S1. Primers for qRT-PCR. Related to STAR methods.**

| Gene | Forward | Reverse |
| --- | --- | --- |
| MLPH | gtcgaatgtcatcaggaatg | gatctgcatctcagaagacg |
| RAB27A | ggaatcccctactttgaaac | attcgcttcattatcaggtc |
| MYO5A | cagagatgaacgaaatcagtc | gtatcgcatggcatacttag |
| GLUL | cctgcttgtatgctggagtc | gatctcccatgctgattcct |
| ATF4 | aaacctcatgggttctccag | ggcatggtttccaggtca |
| PLCG1 | agttccttcttgactaccag | actcatccaggaagaagtatg |
| PLCG2 | tccaccacggtcaatgtagat | ccctgggcggatttcttttat |
| PLCB1 | ggaagcggcaaaaagaagctc | cgtcgtcgtcactttccgt |
| PLCD1 | taagcaggtacccagtgcctggggatccggagc | taagcagcggccgcctagtcctggagggagatc |
| PLCD3 | caagcttatgctgtgcggccgctgga | cggatcctcaggagcgctggatgcgat |
| PLCE1 | tgcagcctctcatccagtt | ccctgcggtaaatatctgc |
| CDH1 | ccgagagctacacgttc | tcttcaaaattcactctgcc |
| CDH2 | acatatgatgaccgtaac | tttttctgatcaagtccag |
| FN1 | ccatagctgagaagtgttttg | caagtacaatctaccatcatcc |
| SNAI1 | ctctaatccagagtttaccttc | gacagagtcccagatgag |
| SNAI2 | cagtgattatttccccgtatc | ccccaaagatgaggagtatc |
| TJP1 | ttgtcttcaaaaactcccac | gactcacaggaataggtttag |
| VIM | ggaaactaatctggattcactc | catctctagtttcaaccgtc |
| ZEB1 | aaagatgatgaatgagagtc | taacttttcatcatgaccac |
| ZEB2 | atttcagggagaattgcttg | tgttcgtatttatgtcgcag |
| TWIST1 | ctagatgtcattgtttccagag | ccctgtttctttgaatttgg |
| MMP2 | gtgatcttgaccagaatacc | gccaatgatcctgtatgtg |
| MMP9 | aaggatgggaagtactgg | gcccagagaagaagaaaag |
| GAPDH | tcggagtcaacggatttg | caacaatatccactttaccagag |

**Table S2. Baseline characteristics of pancreatic cancer patients included in the study.**

| **Characteristics** | **Total (N=97)** | **%** |
| --- | --- | --- |
| **Age, years (median, range)** | 66.0 (37.5-86.0) | |
| **Gender** |  |  |
| Male | 47 | 48.5 |
| Female | 50 | 51.5 |
| **Tumor location** |  |  |
| Head/neck | 70 | 72.2 |
| Body/tail | 27 | 27.8 |
| **Stage** |  |  |
| I | 17 | 17.5 |
| II | 60 | 61.9 |
| III | 13 | 13.4 |
| IV | 7 | 7.2 |
| **Tumor stage (T)** |  |  |
| T1+T2 | 35 | 36.1 |
| T3+T4 | 62 | 63.9 |
| **Lymph Node stage (N)** |  |  |
| N0 | 39 | 40.2 |
| N1 | 58 | 59.8 |
| **Distant metastasis stage (M)** |  |  |
| M0 | 90 | 92.8 |
| M1 | 7 | 7.2 |
| **Tumor size (cm)** |  |  |
| ≦3 | 44 | 45.4 |
| >3 | 53 | 54.6 |
| **Resection margin** |  |  |
| R0 | 81 | 83.5 |
| R1 | 16 | 16.5 |
| **Tumor differentiation** |  |  |
| Well differentiated | 9 | 9.3 |
| Moderately differentiated | 73 | 75.3 |
| Poorly differentiated | 15 | 15.5 |
