# Supplementary material for: Melanophilin-induced primary cilia promote pancreatic cancer metastasis
Source: Cell Death Dis. 2025 Jan 16;16(1):22. doi: 10.1038/s41419-025-07344-2 (PMC11739566; doi:10.1038/s41419-025-07344-2)

Figure 1F

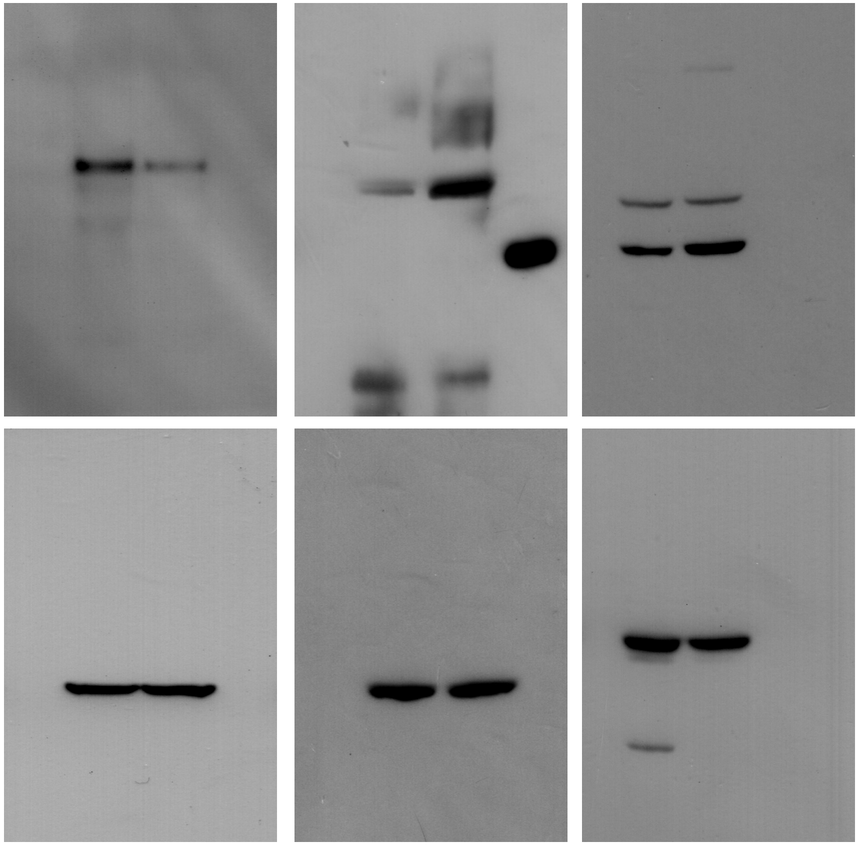

Figure 1H

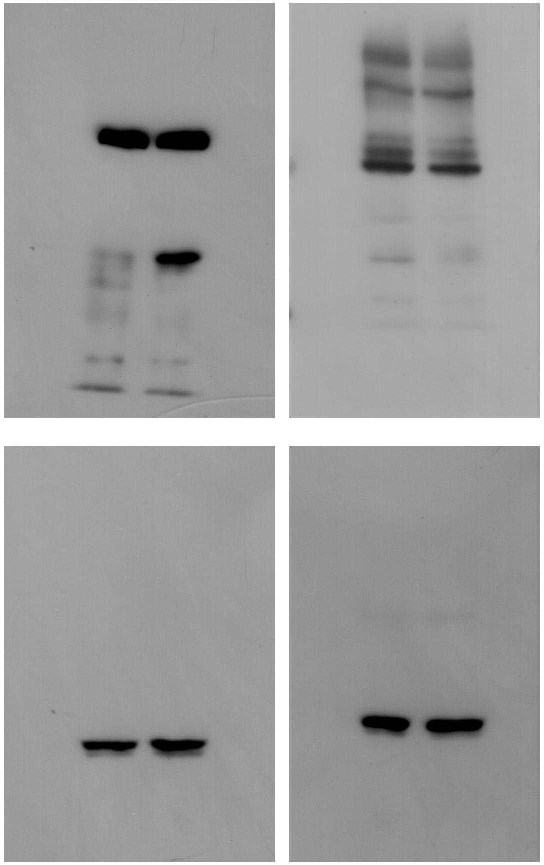

Figure 1I

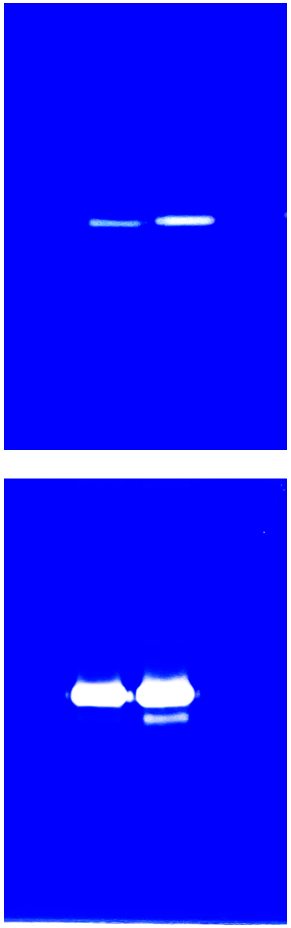

Figure 2C

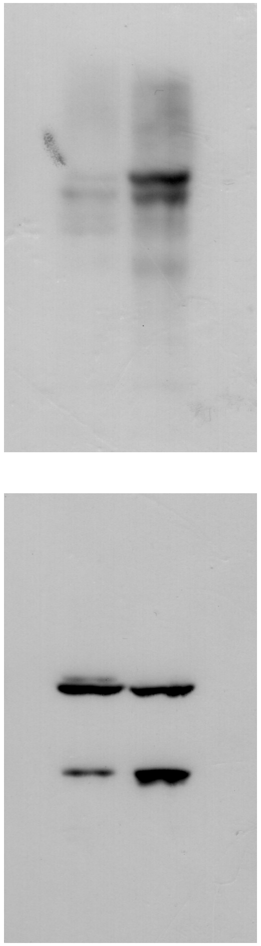

Figure 3A

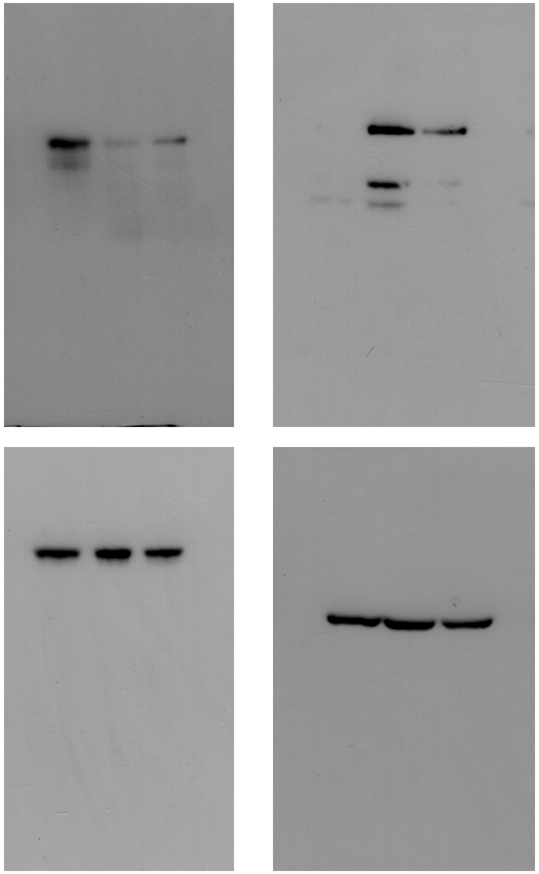

Figure 5G

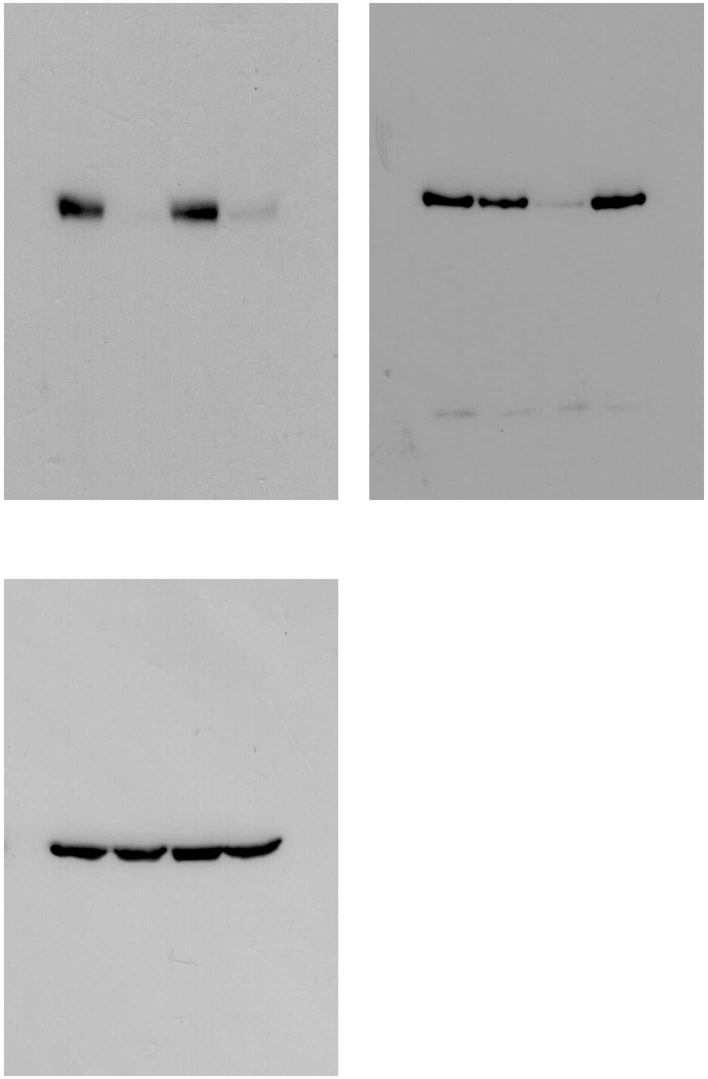

Figure 7A

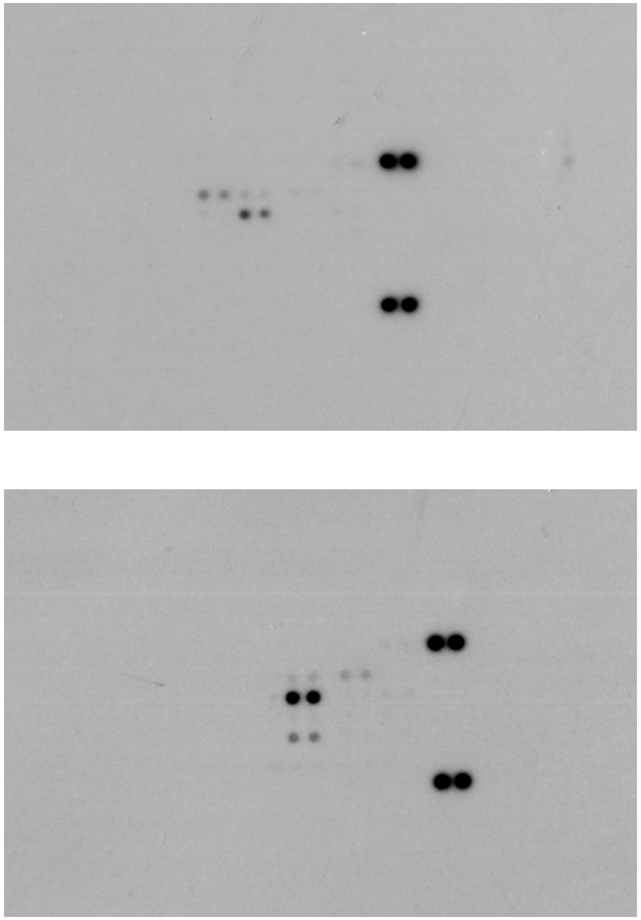

Figure 7B

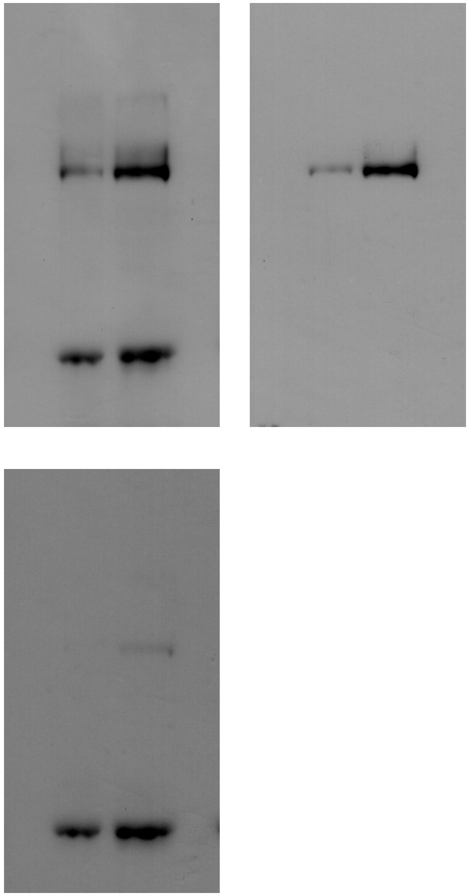

Figure 7D

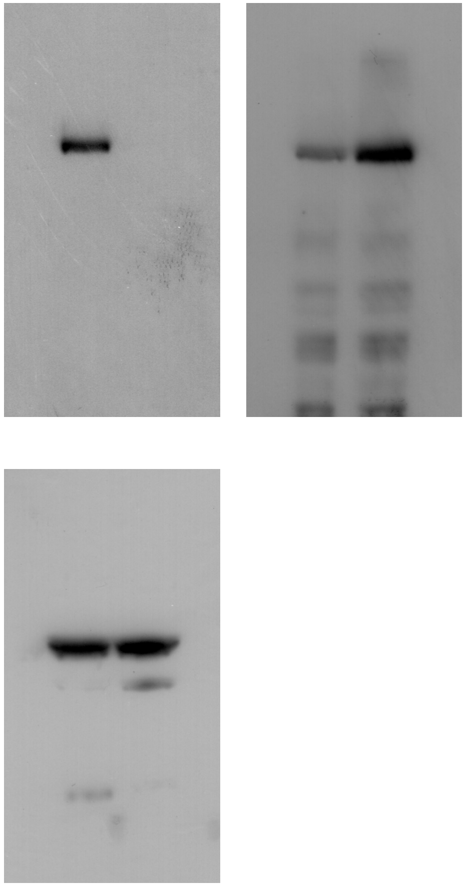

Figure 7G

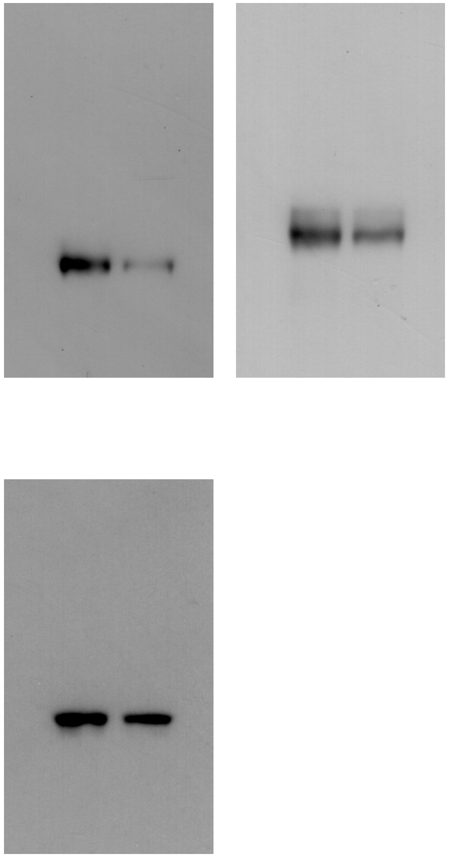

Figure 7H

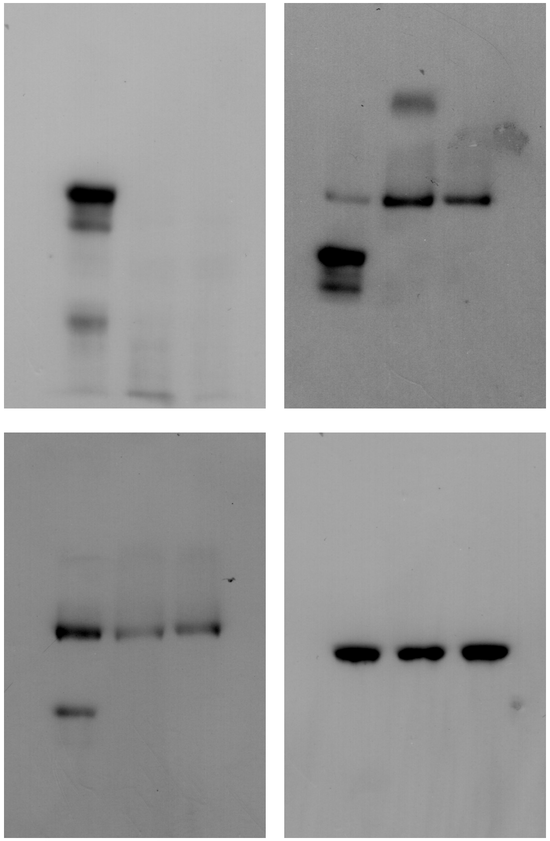

Figure S1G

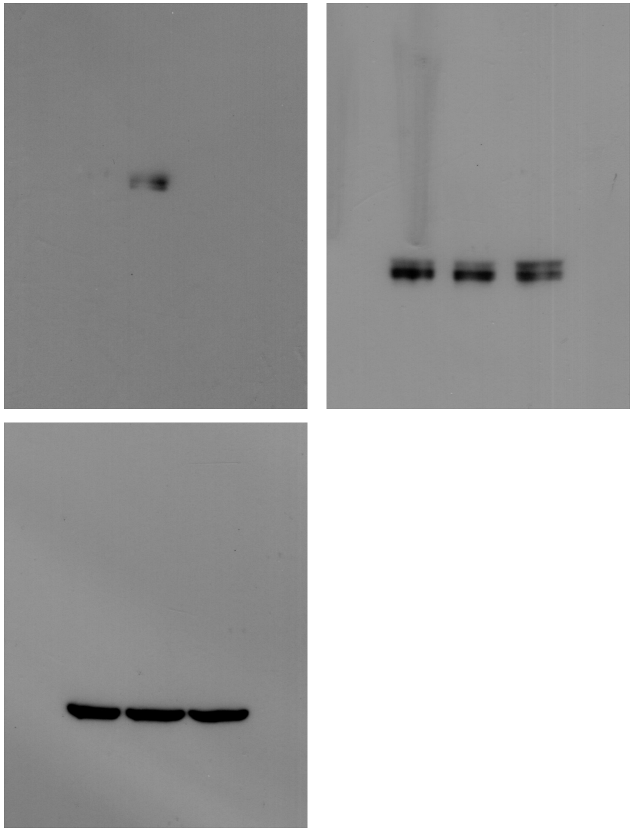

Figure S2A

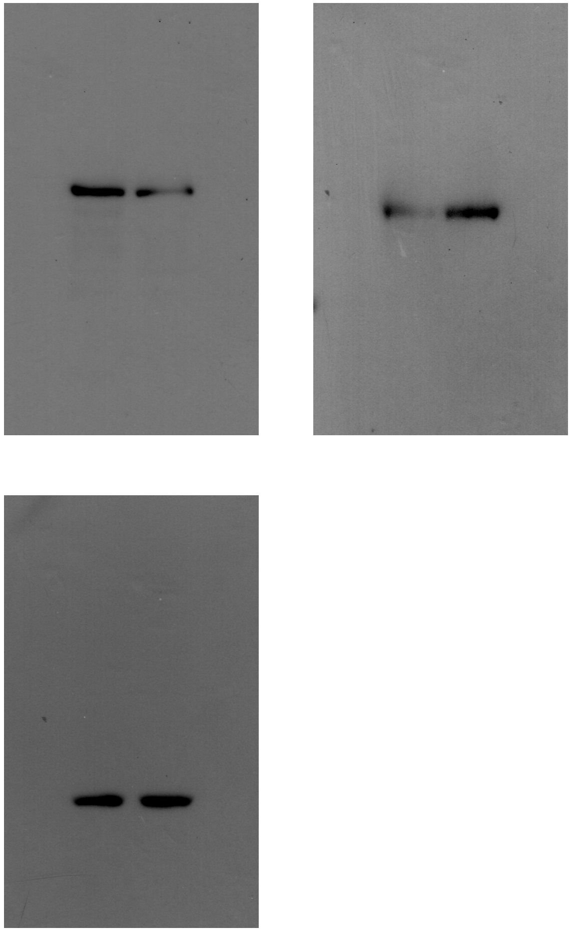

Figure S2C

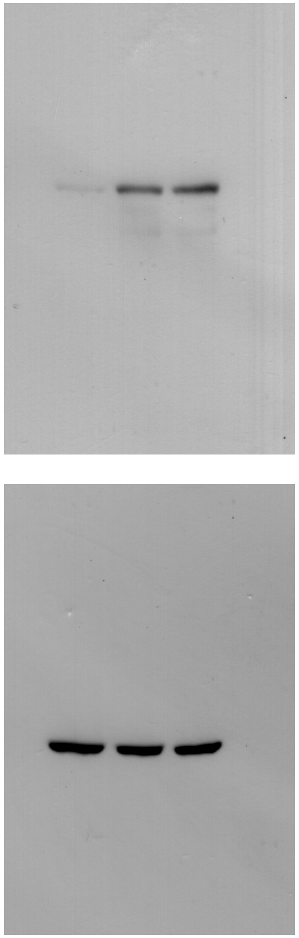

Figure S4A

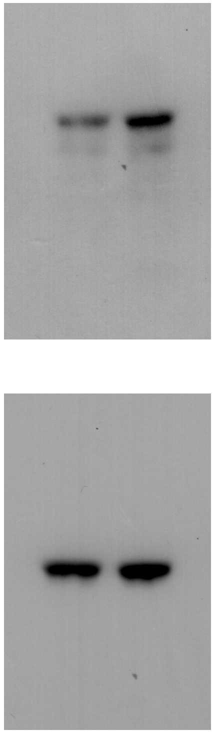

Figure S4B

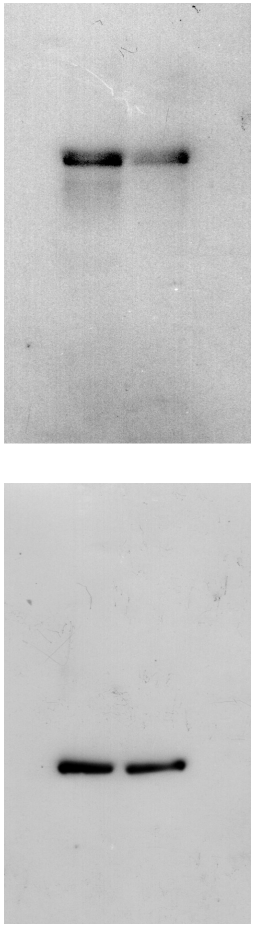

Figure S4C

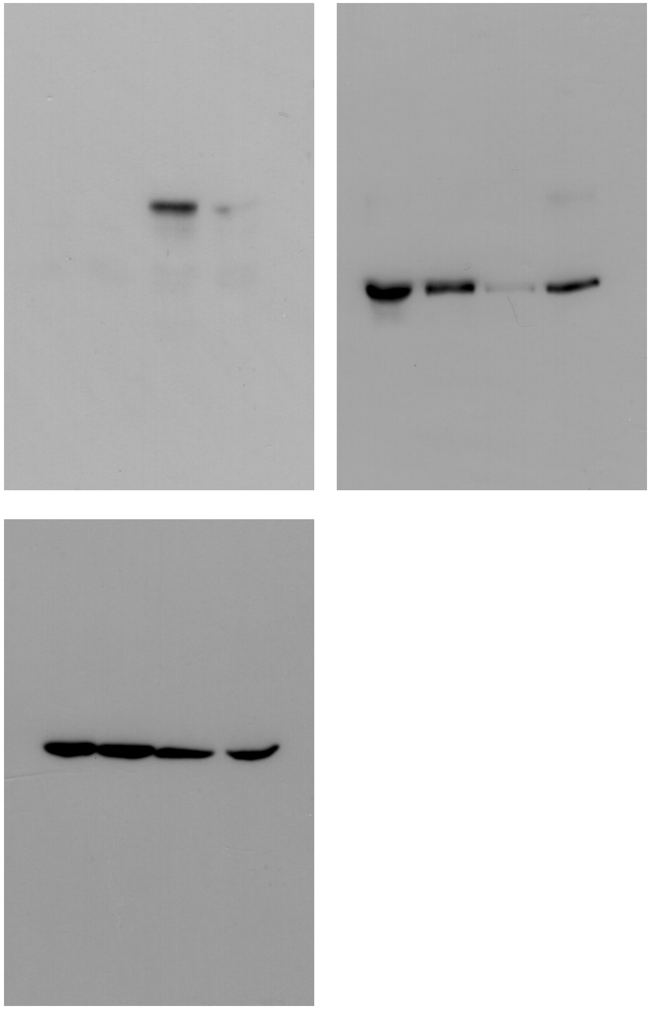

Figure S5F

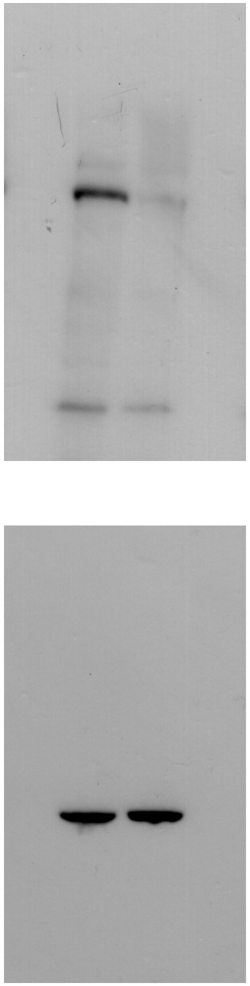

Figure S5H

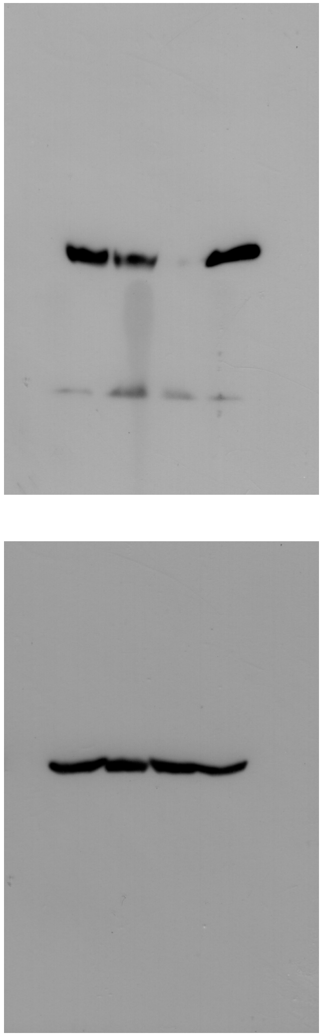

Figure S6A

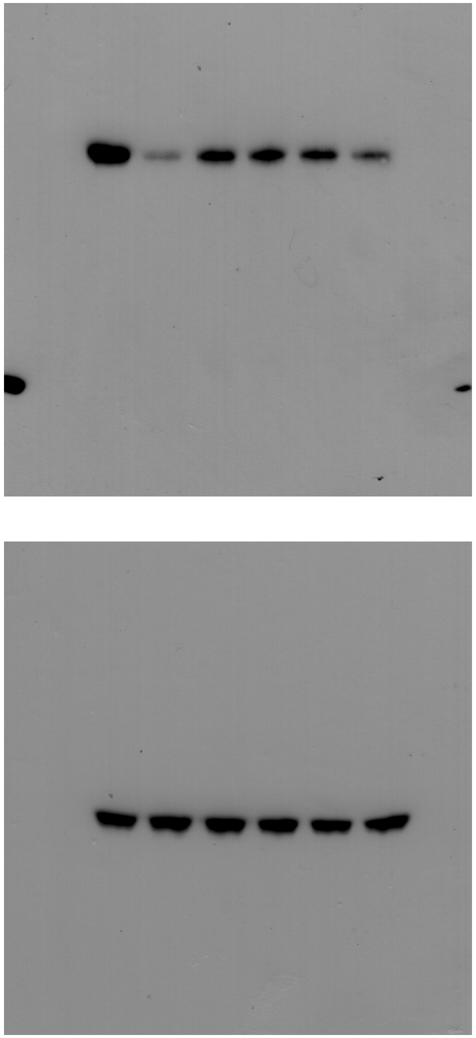

Figure S7A

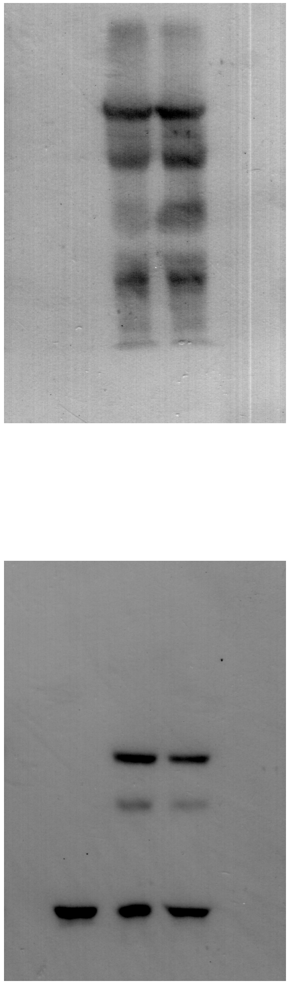

Figure S8B

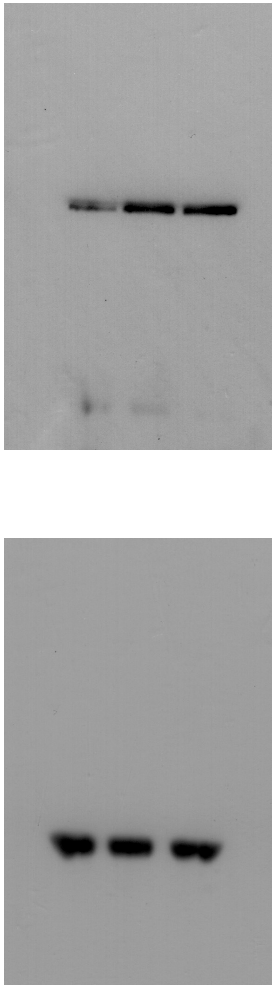

Figure S8E

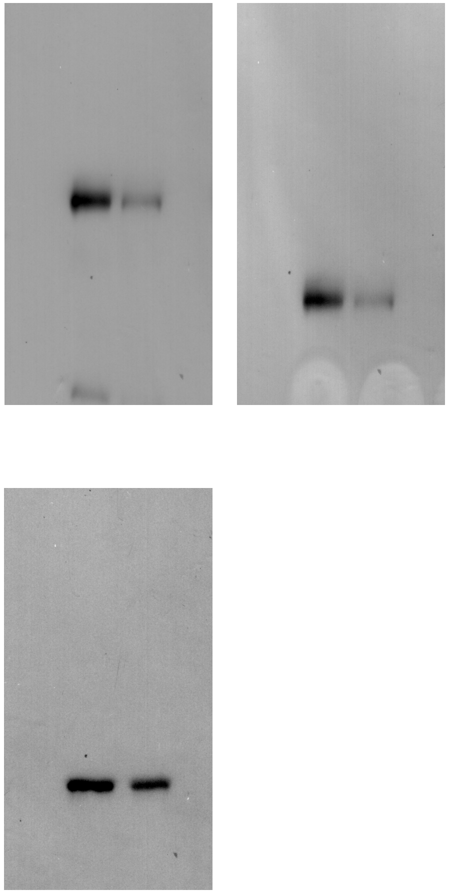

Supplement: Supplementary file 2 — Original data [file 41419_2025_7344_MOESM2_ESM.pdf]
